# Selfishness driving reductive evolution shapes interdependent patterns in spatially structured microbial communities

**DOI:** 10.1038/s41396-020-00858-x

**Published:** 2020-12-20

**Authors:** Miaoxiao Wang, Xiaonan Liu, Yong Nie, Xiao-Lei Wu

**Affiliations:** 1 College of Engineering, Peking University, 100871 Beijing, China; 2 Institute of Ocean Research, Peking University, 100871 Beijing, China; 3 Institute of Ecology, Peking University, 100871 Beijing, China

**Keywords:** Microbial ecology, Microbial ecology

## Abstract

Microbes release a wide variety of metabolites to the environment that benefit the whole population, called public goods. Public goods sharing drives adaptive function loss, and allows the rise of metabolic cross-feeding. However, how public goods sharing governs the succession of communities over evolutionary time scales remains unclear. To resolve this issue, we constructed an individual-based model, where an autonomous population that possessed functions to produce three essential public goods, was allowed to randomly lose functions. Simulations revealed that function loss genotypes could evolve from the autonomous ancestor, driven by the selfish public production trade-off at the individual level. These genotypes could then automatically develop to three possible types of interdependent patterns: complete functional division, one-way dependency, and asymmetric functional complementation, which were influenced by function cost and function redundancy. In addition, we found random evolutionary events, i.e., the priority and the relative spatial positioning of genotype emergence, are also important in governing community assembly. Moreover, communities occupied by interdependent patterns exhibited better resistance to environmental perturbation, suggesting such patterns are selectively favored. Our work integrates ecological interactions with evolution dynamics, providing a new perspective to explain how reductive evolution shapes microbial interdependencies and governs the succession of communities.

## Introduction

Microbes rarely live in isolated niches naturally, but interact with other individuals to form complex communities. It is widely believed that microbial interactions are central to the maintenance, stability, and productivity of these communities [1–3]. Although research has been focused on the evolution of different forms of microbial interactions and their impact on the fitness of the individuals, how the interplay among different individuals governs the succession of microbial communities in the process of long-term evolution has received less attention [4, 5]. Due to the wide range of uncertainty involved in evolutionary dynamics, with the given initial biotic and abiotic components, it is challenging to predict what kinds of genotypes could evolve and which type of interaction pattern could organize in the future community [6, 7]. Resolving this problem is not only important for understanding the formation and maintenance mechanism of microbial diversity, but also has implications for the evolutionary responses of the community to novel environments [8, 9].

One important form of microbial interaction is cooperation related to the production and exchange of so-called ‘public goods’. Public goods are products that, while costly to produce, provide a benefit to all the members of a community, especially to neighbors of the producer [10]. Many secretions released by microorganisms can be considered public goods, such as degradative enzymes [11, 12], siderophores [13–15], detoxification agents [16], and amino acids [17, 18]. Public goods sharing creates an opportunity for the evolution of cooperative interactions. The recently proposed Black Queen Hypothesis (BQH) explains how public goods dynamics drive the origin of dependencies over an evolutionary timescale, predicting that when an individual loses a costly, leaky function, it will receive a selective advantage and expand in the community until the production of public goods is just sufficient to support the equilibrium community [19]. According to the BQH, the origin of cooperative interactions may be based on the selfish trade-off of public goods production by individuals [19, 20]. The BQH has been widely applied to explain the evolution of metabolic dependencies through adaptive functions loss, both for free-living [16, 21–24] and host-associated [13, 21, 25] organisms. However, in complex ecosystems, microbes can exchange a variety of public goods, so multiple functions may be lost through reductive evolution, resulting in diverse ecological outcomes. Could diverse types of interaction patterns, especially the cross-feeding interdependent pattern, emerge from originally autonomous genotypes who can produce more than one public goods through Black Queen evolution? What decides the formation of different interaction patterns? Resolving these questions can help us understand how public goods exchange interactions govern the assembly and succession of microbial communities.

Because of the difficulties in simulating long-term evolution and complicated natural conditions in experimental systems, some studies have applied mathematical modeling approaches to test the potential for cooperation driven by Black Queen evolution. Simulations initialized with diverse cooperative genotypes suggest that the emergence of microbial interdependencies only occurs under specific conditions [26, 27], and in some cases, cooperative interactions are associated with reduced productivity of communities and are, therefore, not selectively favored [26]. However, these models rarely considered stochastic events during evolution, for example, the randomness of the time order and positions for the emergence of different genotypes. A recent study by Mas et al. (2016) applied an agent-based model to test the BQH, and successfully simulated the invasion of a loss of function (LOF) genotype to its autonomously ancestral population [28]. These kinds of model (agent-based model or individual-based model, IBM) focused on spatially-structured environments, thus well-described the randomness of spatial positioning of different mutants. When numerous public goods can be secreted, this randomness will add the uncertainly to the evolution of an interdependent pattern because the emerging positions of LOF genotypes are unpredictable. In addition, IBMs focus on the behavior of individuals, thus can well model the occurrence of random mutations at the single-cell level, displaying good predictive power for the effect of random emergence order of different genotypes. Therefore, we used this type of model framework to investigate the complex evolutionary processes driven by public goods sharing and how it shapes microbial community diversity.

Therefore, we proposed that different types of cooperative interdependent patterns could potentially evolve via Black Queen evolution, and constructed an individual-based model to test this idea. We simulated the spatiotemporal dynamics of one type of reductive evolution, starting with an ancestral population that could produce multiple fitness-promoting public goods, who was subsequently allowed to randomly mutate to lose those functions. We focused on investigating the conditions favoring the evolution of different patterns, considering both deterministic factors, such as function cost and functional redundancy, and stochastic factors, such as the randomness of the time order and positions, in our model. We also conducted numerous replicated simulations and genotype lineage tracing to capture the diversity and clustering of evolutionary paths. In sum, we built a new mathematical framework integrating the ecological interactions with evolution dynamics, providing a new perspective to explain how microbial interactions govern the succession of the communities over an evolutionary time scale.

## Results

### The logic of the model

Our spatially-resolved model was simulated in discrete grid boxes of a 100 × 100 array, which included four basic assumptions: (1) Initial individuals were assumed to secrete three public goods but may randomly mutate to lose any of those functions with a certain probability; (2) Secreting a public good created a corresponding metabolic burden, therefore in losing a function the individual would gain a benefit; (3) All public goods were essential for growth. The net growth rates of individuals were dependent on the local concentrations of public goods; (4) Substrate and public goods diffused between two grid boxes at rates proportional to the concentration gradient.

**Fig. 1:**
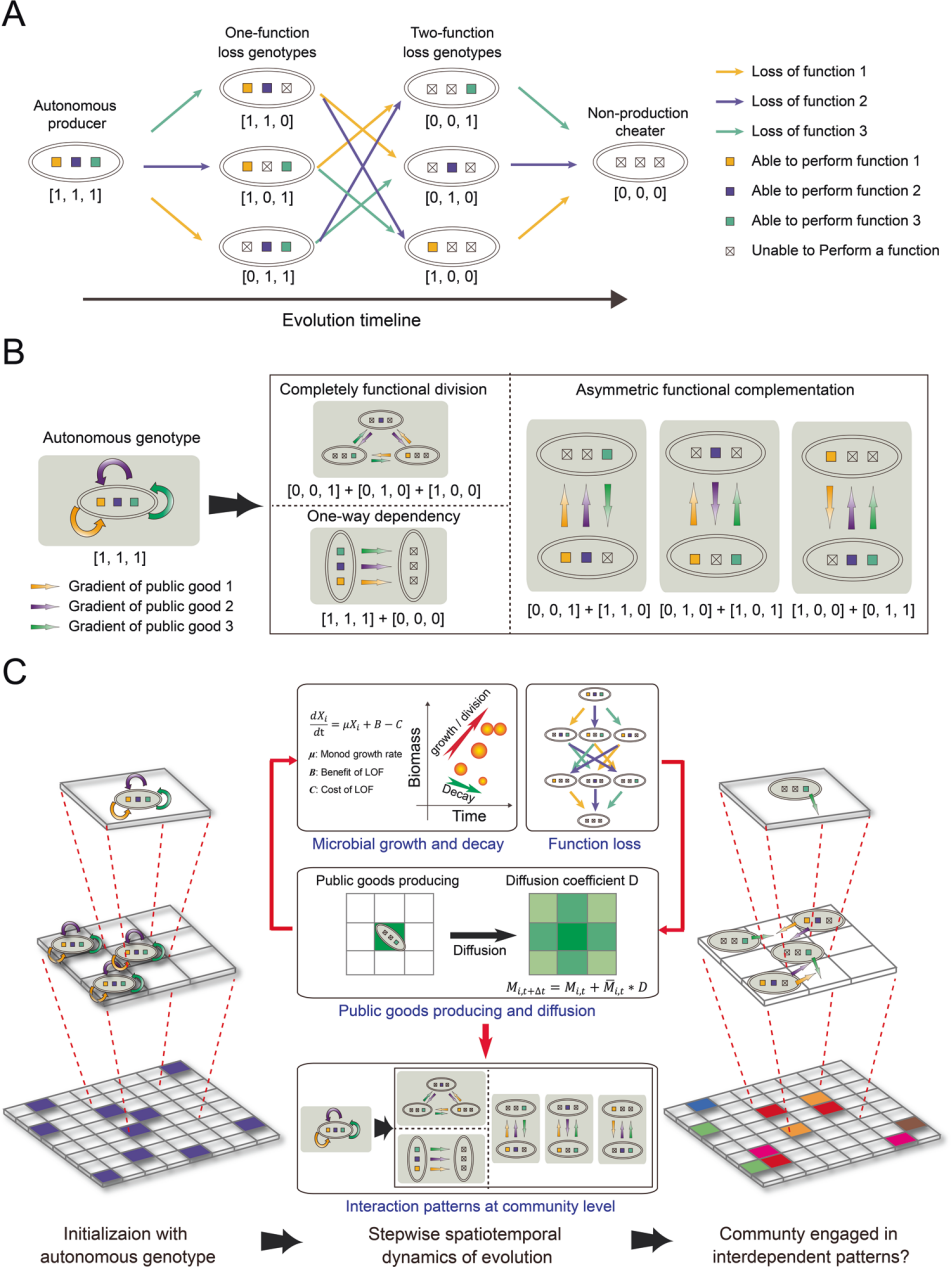
Logic of the individual-based model. **A** Possible genotypes and evolutionary relationships among them emerging from reductive evolution when starting with an autonomous genotype that performs three essential public functions. Note that in this three-function model, some genotypes, i.e., Two-function loss genotypes and cheaters, might evolve from different mother genotypes. **B** Interaction patterns that could possibly be established in the spatially structured communities. **C** Schematic of the individual-based simulations. A 100 × 100 array initialization with all autonomous phenotypic individuals (left) was conducted with a long-term stepwise iteration to investigate if diverse interaction patterns could form (right). At each time step, calculations were done from the level of individual grids (top) to whole lattice (bottom). Within each grid box, Monod equation modified by basic assumptions of the Black Queen Hypothesis was used to calculate the microbial growth, while minimum and maximum thresholds of biomass were defined to decide the division and death of individuals (top middle). Microbial individuals were allowed to randomly mutate to lose functions (top middle). Classical discretization of the diffusion equation gave local rules for updating the concentrations of public goods and nutrients in each box (middle). State changes at the individual level lead to the evolutionary dynamics of the communities, which may give rise to the formation of diverse interaction patterns (bottom).

For the 1st assumption, we included three functions because it is the minimal unit and tersest design to simulate complex communities, allows for the emergence of three categories of interaction patterns, and a single cooperative LOF genotype might evolve from differential evolutionary paths (Fig. 1A, B). The genotypes were described by bit strings containing 1 and 0 which indicated the genotype could produce the corresponding public good or not, respectively. Eight genotypes could emerge during the simulations, which were the initial autonomous producer [1, 1, 1], three one-function loss genotypes (OFLGs, i.e., [1, 1, 0], [1, 0, 1], and [0, 1, 1]), three two-function loss genotypes (TFLGs, i.e., [1, 0, 0], [0, 1, 0], and [0, 0, 1]), and a nonproducing cheater [0, 0, 0] (Fig. 1A).

The 2nd and 3rd assumptions were developed from the basic mathematical assumption of the BQH [19], and defined individual growth by integrating the benefit and cost of function loss (Fig. 1C). To conceptualize the cost of performing a function, we supposed a parameter (*α*) which is the fraction of biomass used to produce a public good per unit time of an individual. In addition, we defined a second parameter (*β*) as the ratio of the amount of public goods required during each step to account for the produced public goods. Therefore the redundant fraction of public goods production was 1−*β*_*j*_, and lower *β*_*j*_ reflected a higher amount of redundant public goods that could be gained from the producers by the LOF genotypes, resulting in decreased risk in association with function loss (see Supporting Information S1 for more details). During the model simulation, spatiotemporal dynamic variables, i.e., positioning of genotypes and the time points at which genotypes evolved, would be collected. We initiated the simulations by randomly distributing 100 ancestor cells [1, 1, 1] into the grid boxes and iterated for at least 1,500,000 time steps. During each time step, individuals grew, decayed, reproduced, and mutated according to the previously mentioned assumptions (Fig. 1C). We paid attention to whether stable communities with various interdependent patterns could be formed after a specified number of iterations, as well as recorded the spatiotemporal dynamics of the communities.

### Diverse interdependent patterns emerged with high level of function cost and varied level of functional redundancy

**Fig. 2:**
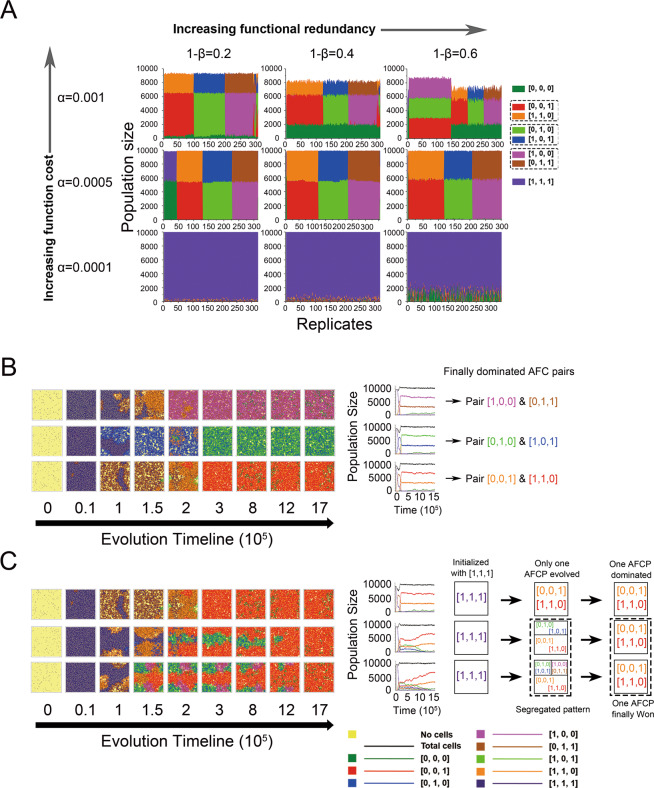
Reductive evolution shapes diverse interdependent patterns in microbial communities. **A** The final (steady state) community structures across gradients of function cost (α) and functional redundancy (1-β). Results were summarized from at least 300 interdependent runs for each parameter set. Community structures were assessed after simulation for 170,000 iterations, where 98.9% (2891/2923) of runs reached steady state. According to the structures, replicates were clustered into several scenarios for each parameter set, which are shown separately in the area plots. Note that the values of β is the proportion of public goods that is required for growth, and thus 1-β reflects the level of function redundancy. **B**, **C** Six representative community dynamics on the spatial lattices were selected from one interdependent simulation with the given conditions (*mut* = 10^−5^, α = 0.001, β = 0.8), showing the evolution of three types of asymmetric functional complementary pairs (AFCPs) (**B**), three different paths for the evolution of pairs [0, 0, 1] & [1, 1, 0] (**C**). Left images indicate the distribution of different genotypes at different points in evolutionary time. Curve plot in the middle describes the community dynamics of the corresponding simulation. Schematics at right briefly summarize the spatiotemporal dynamics of each simulation: the arrays in (**B**) indicate one type of AFCP directly dominated the communities without competition from others; the boxes in (**C**) indicates the composition of ancestor or AFCP in the related time points, while the windows inside indicate the spatial coexistence of multiple AFCPs and the size of the windows represents the relative fraction of different AFCPs.

For model simulations, the function cost (parameter α) and functional redundancy (parameter β) were assigned to 0.0001, 0.0005, 0.001, and 0.4, 0.6, 0.8, respectively. A total of 2891 independent simulations with 9 parameter sets displayed different community structures (Fig. 2A). When the function cost was assigned to a low level, i.e., 0.0001, the autonomous ancestor dominated the community. When function costs were assigned to higher levels, 0.0005 and 0.001, new genotypes evolved and later interacted to form three distinct types of interdependent patterns even within the same α and β combination, i.e., asymmetric functional complementation (AFC), complete functional division pattern, and one-way dependency, with the relative amounts of 1677/2891, 143/2891, and 48/2891, respectively. In addition, higher functional redundancies favored the loss of more functions, increasing complexity of the community structures.

Among the three possible kinds of interactions, the AFC pattern was the most widespread, which was the combination of a two-function-loss genotype (TFLG) and its complementary one-function-loss genotype (OFLG). For example, [0, 0, 1], which produced a single essential public good, depended on its functional complement one-function-loss partner [1, 1, 0], for the other two public goods. Specifically, three types of the asymmetric functional complementary pairs (AFCPs), that is, [0, 0, 1] coupled with [1, 1, 0], [0, 1, 0] coupled with [1, 0, 1], and [1, 0, 0] coupled with [0, 1, 1], colonized most of the grid with a similar frequency of emergence. Interestingly, under the condition of high level of cost, the emergence of AFC patterns was accompanied by some nonproducing cheaters, whose relative abundance rose with the increase in functional redundancy (Fig. 2A top row). The addition of cheaters significantly reduced the total biomass of the communities, suggesting that high functional redundancy favors the evolution of cheaters which may decrease the community productivity. In addition, function loss happened more easily with high function cost. As the function cost parameter α increased from 0.0005 to 0.001, relative abundance of TFLGs increased approximately from 55 to 70% (Fig. 2A).

Besides the AFC patterns, two additional types of interdependent patterns evolved at a relatively lower frequency. The complete functional division pattern, that is, coexistence of [0, 0, 1], [0, 1, 0], and [1, 0, 0], only evolved when both factors were at high levels (*α* = 0.001, *β* = 0.4) with a frequency of approximately 45% (143 of 319 simulations, Fig. 2A, top right), which described a scenario with high benefit and low cost of function loss, favoring the loss of more functions and consequently more likely to maintain the evolution of TFLPs. Another form of interactions that emerged was one-way dependency, where one partner performs all functions and other none (i.e., coexistence of [1, 1, 1] and [0, 0, 0]). This form emerged at a low frequency (48 out of all 2891 simulations shown in Fig. 2A), but evolved with a higher probability under the condition of a mid-level function cost and low level of functional redundancy (*α* = 0.0005, *β* = 0.6, Fig. 2A, middle left), where the extinction of [1, 1, 1] was ~2.5 times slower than in other scenarios (Supplementary Fig. 1), leading to a higher potential for the spatial proximity between [1, 1, 1] and [0, 0, 0] during evolution.

Taken together, these phenomena demonstrated that the mutualistic exchange of complementary functions happened only when function cost was high. The emergence of different interdependent interaction patterns was related to the function cost and function redundancy, especially for the complete functional division and one-way dependency pattern, which only emerged within a limited parameter range. However, even for a given combination of α and β, it still remained possible for the evolution of distinct interaction patterns, suggesting that stochastic processes may play a role.

### Same interdependent patterns might evolve via different modes

Because the evolution of three kinds of AFCPs were the most common scenarios in our simulations, we then focused on the role of stochastic processes, i.e., the key random events, in deciding the winning complementary pair among the three similar but different AFCPs. As a first step, we traced the variation in the spatiotemporal dynamics, trying to cluster the numerous evolutionary dynamics into limited modes and divide the complex evolutionary courses into several stages. These simplifications would facilitate the search for key random events.

Therefore, we analyzed the dynamics of 296 simulations with a typical parameter set (*α* = 0.001, *β* = 0.8), because under this condition, only the three types of AFCPs evolved, with a similar frequency of emergence (Fig. 2A, Top left), in order to avoid interference from the other interaction patterns. As described above, any of the three types of AFCPs could potentially take over the final community under this condition (Fig. 2B; Supplementary video 1–3). Using the emergence of AFCP [0, 0, 1] & [1, 1, 0] as an example, three categories of dynamic modes could give rise to its final domination. (1) After pair [0, 0, 1] & [1, 1, 0] emerged and formed a spatial aggregation, it rapidly expanded and took over the entire grid (Fig. 2C, first line; Supplementary video 3). (2) In addition to the pair [0, 0, 1] & [1, 1, 0], spatial aggregations of another AFCP also emerged (e.g., pair [0, 1, 0] & [1, 0, 1] in Fig. 2C, second line and Supplementary video 4). In this scenario, a special spatial pattern was established in a short period after the evolution of both AFCPs e, where pairs of two complementary members exhibited strong spatial mixing, while the two different AFCPs were totally segregated. Community succession was then governed by spatial competition between the two AFCPs. If pair [0, 0, 1] & [1, 1, 0] won the competition, it would dominate the final community. (3) Spatial aggregations of all three AFCPs emerged, and then pair [0, 0, 1] & [1, 1, 0] dominated the community after outcompeting the other two AFCPs (Fig. 2C, third line; Supplementary video 5). The clustering of these three possible modes of AFC patterns was also shown by the temporal dynamics of the α-diversity across different parameter sets (Supplementary Fig. 2), where the evolution modes of the AFC patterns were clearly clustered into three possible categories, suggesting that this clustering is independent of the determined factors α and β.

In sum, the succession of interdependent patterns could be divided into two stages: (1) the emergence of spatial aggregations composed of two interdependent members with strong connections; (2) spatial competition among different aggregations drive the community to evolve to the final state, composed of only one type of interdependent interactions. Of course, if only one type of AFCP emerged, the spatial competition stage would be unnecessary during succession.

### Evolutionary random events play important roles in deciding the dominant AFCP in equilibrium communities

The presence of two evolutionary stages lead us to hypothesize that the random events affecting ecological outcomes should arise from two aspects. First, in the initial evolutionary stage, the emergence of interdependent spatial aggregations should be related to the order in which new genotypes emerge. Second, the outcome of the spatial competition should be also influenced by the initial positioning of the new genotypes.

**Fig. 3:**
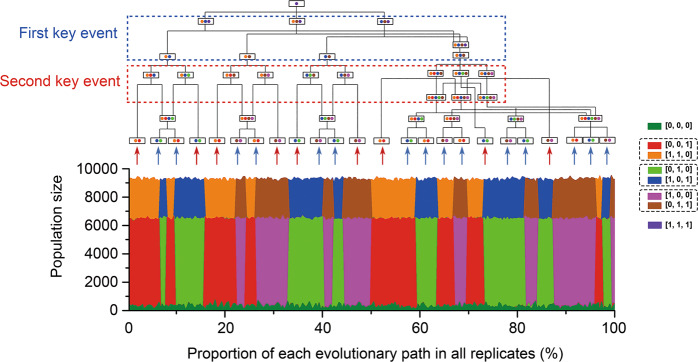
The evolutionary trajectories of 296 independent simulations with the typical parameter set (*mut* = 10^−5^, *α* = 0.001, *β* = 0.8). We analyzed the evolutionary trajectories of every interdependent run and clustered them into 24 types of branches (top, see Methods). The area plot shows the final community structures and the frequencies of each branch (bottom). Blue dashed box shows the evolutionary diversification into four scenarios after the first key event occurs, while the red dashed box indicates the 24 different evolutionary trajectories that diverged after the second key event occurs. Solid boxes with colored circles represent the genotypic composition of communities at different evolutionary time points. Red arrows indicate the branches where one type of asymmetric functional complementary pair (AFCP) directly dominated the communities without competition with other AFCPs, while the blue arrows indicate the branches where one type of AFCP took over the entire space after competitions with other AFCPs. Dashed boxes at the figure labels (right) indicate different AFCPs.

The fact that each TFLP had two possible evolutionary paths (e.g., [1, 0, 0] could inherit its function from [1, 1, 0] or [1, 0, 1]), suggested that the effects of the random order of emergence for different genotypes were highly correlated with the evolutionary lineage. Therefore, to investigate the effects of this, we analyzed the evolutionary lineage of emergence, colonization, and loss of every genotype within the 296 simulations with the typical parameter set (*α* = 0.001, *β* = 0.8). In total, there were 24 evolutionary branches leading to the evolution of the three forms of AFC patterns (8 for each, Fig. 3). Among all these branches, we summarized two key random events (Fig. 3, red and blue boxes).

The first event occurred after two types of OFLGs emerged. After this evolutionary time point, all three public functions were included in OFLGs. With the benefit of the function loss, these two OFLGs would expand and gradually outcompete the autonomous genotype [1, 1, 1]. Thus, the first key event was whether all three OFLGs could emerge before the autonomous genotype entirely disappeared (Fig. 3, blue box). If not, the third type of AFCP would never evolve; if so, all three types of AFCPs would still have a chance to dominate the final community. In the 296 simulations, the frequencies of these two scenarios were nearly same, that is, 147 simulations were clustered to the former, while 149 simulations were clustered to the latter. The 147 simulations, where the third type of AFCP never evolved, could be then divided into three categories with similar frequencies, where two of the three OFLGs occupied the whole space and excluded the ancestral population.

The second key evolutionary event was the emergence of TFLGs (Fig. 3, red box). After the two or three types of OFLGs successfully colonized, whose functional complementary TFLGs first to emerge in the next evolutionary time would lead to the prior formation of the spatial aggregation of the AFCP. It is obvious that if no other AFCP aggregations formed later, this AFCP would dominate the final community (Fig. 3, red arrow indicated branches). Alternatively, if other AFCP aggregations formed during the expansion process, the spatial competition between different AFCPs would decide the dominant AFCP in the equilibrium communities (Fig. 3, blue arrow indicated branches). In our analysis, the chance of only one AFCP evolving reached 64.7% (198 of the 296 simulations). If only two OFLGs evolved after the first event, the frequency of only one AFCP evolving reached 79.6% (121 of the 152 simulations). In contrast, if three OFLGs evolved after the first event, there could be a relative higher possibility of two or three AFCPs evolving (47.4%), meaning that spatial competition could then be an important process.

**Fig. 4:**
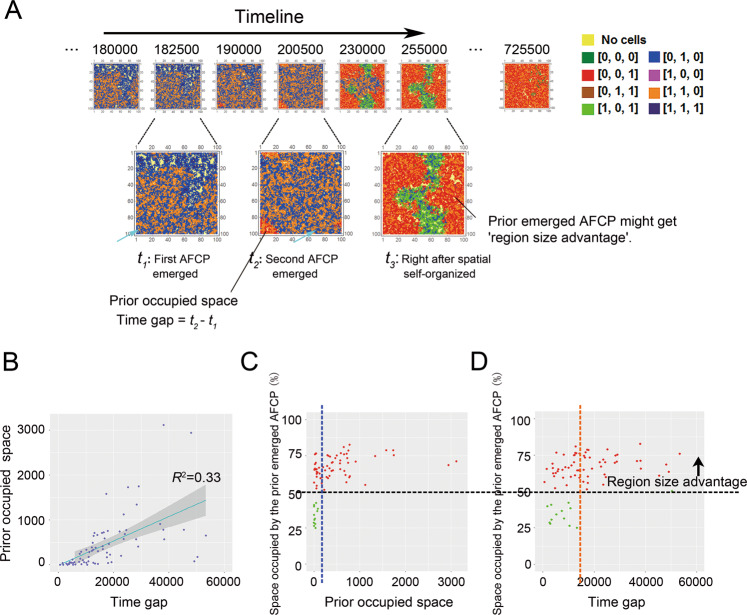
The asymmetric functional complementary pair (AFCP) that emerged first would obtain a ‘region size advantage’ to help it win in competition against other AFCPs. **A** A typical example of simulations was selected to illustrate the development of the segregated interdependent spatial pattern. After the successful colonization of [1, 1, 0] and [1, 0, 1], Two-function loss genotype (TFLG) [0, 0, 1] emerged first and gathered with its functional complementary partner [1, 1, 0] to form a spatial aggregate, and occupied some space. After a TIME GAP, the second TFLG [0, 1, 0] also emerged, aggregated with its partner [1, 0, 1], and expanded. Due to the prior space occupancy, the aggregate formed by [0, 0, 1] and [1, 1, 0] can occupy more space, possessing a ‘region size advantage’, which help it to win the competition that followed between the aggregates. Cyan arrows indicate the position of the emergence of the two TFLGs. **t**_**1**_: time point when the TFLG member of the first AFCP emerged. **t**_**2**_: time point when the TFLG member of the second AFCP emerged. TIMEGAP equals **t**_**2**_ - **t**_**1**_, that is, the time lag between the emergences of two TFLG members of the two AFCPs. **t**_**3**_: time point when the segregated interdependent spatial pattern was newly established, which was defined as the first time when the total abundance of the two AFCPs reaches 95%. **B** Correlation between the prior occupied space sizes of the first emerged AFCP at t_2_ and the TIME GAP. **C** Percentage of the community space occupied by the first emerged AFCP at t_3_ as a function of its prior occupied space size at t_2_. **D** Percentage of the community space occupied by the first emerged AFCP at t_3_ as a function of TIMEGAP. For (**B**), (**C**) and (**D**), results were analyzed from all replicates, where two types of AFCPs formed a segregated spatial pattern during the evolution (75 replicates, from 296 interdependent simulations related to Fig. 3). Red dots indicate the first emerged AFCP won the competition in the corresponding replicate, while, conversely, the green dots indicate the second emerged AFCP won the competition. Blue dashed line in (**B**) indicates a dividing value of prior occupied space size, i.e., when prior occupied space size over that value, the first emerged AFCP would surely win the competition. Orange dashed line in (**C**) indicates the dividing value of TIMEGAP, i.e., over this value the first emerged AFCP would surely win the competition.

What decided the winner of the competition? We observed that after the segregated interdependent spatial pattern was newly established, the relative region sizes occupied by different AFCPs were the key to determining the winner (Fig. 2C, the second and third lines; Supplementary video 4 and 5). We analyzed the time gaps between the emergence of the two AFCPs in the second categories of succession modes and the size of the regions they occupied (Fig. 4A). The result indicated a significantly positive correlation between the length of the time gaps and the region size the prior AFCP occupied (*t*-test, *p* < 0.05; Fig. 4B); if the time gap was greater than ~17,000 time steps, or the prior occupied space was larger than 129 grid boxes, the first emerged AFCP would win the competition (Fig. 4C, D). Together with the lineage analysis, these results confirmed that the order of emergence by the different genotypes would largely decide the formation of different interdependent patterns in the final communities, i.e., the earlier an AFCP emerged, the greater the chance for it to dominate the equilibrium communities.

**Fig. 5:**
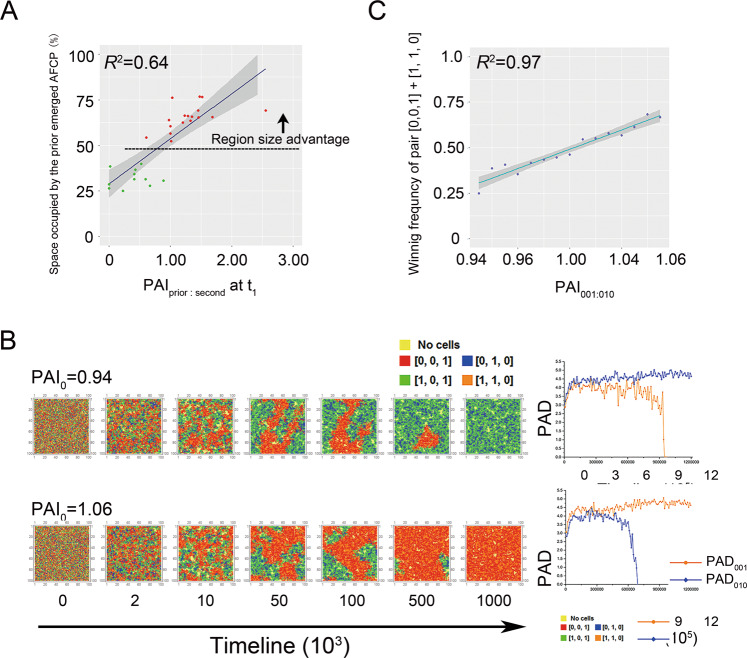
Higher initial spatial association degree of an asymmetric functional complementary pair (AFCP) facilitated its winning. **A** When the two AFCPs evolved nearly simultaneously, the initial relative association degree of the two AFCPs showed significant positive correlation with the outcomes of competition. We selected the replicates in Fig. 4B–D, whose TIME GAP was below the dividing value (see Fig. 4C). PAI_prior:second_ values were calculated from the communities when the TFLG member of the second AFCP emerged (**t**_**2**_, see Fig. 4A), while PAI_prior:second_ > 1 means the prior emerged AFCPs are more spatially associated than the second AFCPs. Red dots indicate the first to emerge AFCP won the competition in the corresponding replicate, while the green dots indicate the second to emerge AFCP won the competition. **B** Two typical examples of simulations initialized with premixing the two types of AFCPs, [0, 0, 1] & [1, 1, 0] and [0, 1, 0] & [1, 0, 1], which represent scenarios when initial PAI_001:010_ < 1 and >1, respectively. **C** The significant positive correlation between the winning frequency of pair [0, 0, 1] & [1, 1, 0] and the initial value of PAI_001:010_. When initial PAI_001:010_ > 1, final communities were more likely to be dominated by pair [0, 0, 1] & [1, 1, 0], oppositely, pair [0, 1, 0] & [1, 0, 1] were more favorable when PAI_001:010_ < 1. Running parameters for (**B**) and (**C**): *mutation rate* = 0, *α* = 0.001, and *β* = 0.8. Simulation protocol of (**C**) was described in Methods section.

Next, we investigated the role of spatial positioning of new genotypes. AFCPs that emerge first would have an opportunity to occupy more space which would favor its winning, but what if different AFCPs evolved simultaneously? Previous results indicated that if there was just a very short time gap between the emergence of the first and second TFLG, the advantage of prior space occupancy was not significant (Fig. 4C). In our simulations, the occasional fortuitous proximity of a focal OFLG cell to its functional complementary partner cell may also benefit the formation of the interdependent spatial aggregate. Therefore, higher association degree at the beginning of self-organization might facilitate space occupation, and help the AFCP gain the ‘region size advantage’ when the segregated spatial pattern is initially established. To test this hypothesis, we defined ‘partner association degree’ (PAD) and ‘partner association index’ (PAI) in a manner similar to a previous study [29], in order to quantify the different relative association degrees of AFCPs. PAD is the average number of the weighted functional complementary partner cell in the immediate neighborhood of a focal TFLG cell (Supplementary Fig. 3A). PAI is the ratio of PADs of the two AFCPs, which quantify the relative PAD between them. For example, PAI_001:010_ equals PAD_001_/PAD_010_, which quantify the relative PAD between pair [0, 0, 1] & [1, 1, 0], and pair [0, 1, 0] & [1, 0, 1], while PAI_001:010_ > 1 indicates pair [0, 0, 1] & [1, 1, 0] is more spatially associated than pair [0, 1, 0] & [1, 0, 1]. Applying these definitions, the simulation results where the advantage of prior space occupancy was not significant (left side of blue line in Fig. 4C, 33 replicates) were selected for analysis, and we found a significantly positive correlation between the relative PAD at the beginning of spatial self-organization and the ‘region size advantage’ (Fig. 5A; *p* < 0.05). This analysis demonstrated that the random spatial proximity between the positions of emergence for two members of an AFCP was important in determining the competition outcome.

To further prove this idea, we conducted additional simulations. Four genotypes, [0, 0, 1], [1, 1, 0], [0, 1, 0], and [1, 0, 1], were inoculated with the same initial number. We randomly distributed the genotypes in the 100 × 100 grids to obtain diverse initial spatial distribution (see Methods; Supplementary Fig. 3B). We then selected a series of distributions with a gradient of relative association degrees of AFCPs (PAI_001:010_), and ran simulations initialized with those values. The spatial self-organization also occurred in these cases, accompanied by the increase of PAD_001_ and PAD_010_ (Fig. 5B; Supplementary Fig. 4). For a given initial distribution, we conducted 100 replicate simulations to calculate the frequency of winning. A strong positive relationship between initial association degree of an AFCP and its winning frequency was found (Fig. 5B, C, *P* < 0.01), which further confirmed the important role of relative positioning on deciding the ecological outcome.

One might ask, in absence of emergence order and relative positioning, whether other random events impacted the outcomes of spatial competition? To address this issue, we simulated communities starting from a symmetric initial distribution of cells, and two types of AFCPs, [0, 0, 1] & [1, 1, 0] and [0, 1, 0] & [1, 0, 1], were equally inoculated (Supplementary Fig. 5A). Eliminating initial spatial asymmetry and distinct space occupancy, the segregated interdependent spatial pattern still emerged, and one of the AFCPs dominated the final community in each run (Supplementary Fig. 5B). However, the frequencies of winning for the two AFCPs were nearly identical in the 100 repeated simulations (Supplementary Fig. 5C), suggesting other random events did not change the relative winning probability of the two AFCPs.

### Interdependent pattern of community is associated with increased stability

**Fig. 6:**
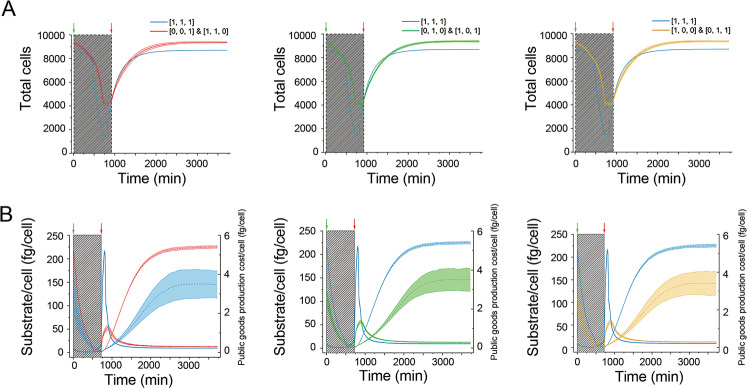
Community dynamics during the nutrient disturbances. Final communities (after 1,700,000 time-step iterations) composed of pair [0, 0, 1] & [1, 1, 0], pair [0, 1, 0] & [1, 0, 1], or pair [1, 0, 0] & [0, 1, 1] were selected to be treated with nutrient disturbances, respectively. As the control, the communities from the same simulations but only after 500 time-step iterations (still dominated by [1, 1, 1]), were taken out to perform same treatments. We stopped the substrate supply for 12 h, then rerestarted the supply. Dynamics of each population are shown in (**A**), where each curve shows the average results of five repeated runs starting with ten representative communities selected from the 296 interdependent simulations mentioned before. The average available substrate for a cell (solid line) and the cost of public goods production (dashed line) at each step during simulations are shown in (**B**). Running parameters: *mutation rate* = 0, *α* = 0.001, and *β* = 0.8.

Do the communities dominated by functional complementary LOF genotypes exhibit distinct community properties, e.g., different community stability? Our mathematical framework allowed us to make comparisons among communities at different time points. To investigate whether community stability changes with the succession, we imposed nutrient disturbances on the communities at initial and final time points. We stopped the nutrient supply for 12 h, and after restoring it, explored community response to the disturbance. Although all communities declined after nutrient depletion, the resistance of the evolved communities to disturbances was better than the original community composed of only the autonomous population, suggesting the evolved communities containing interdependent patterns were more stable (Fig. 6A). The improved community stability was attributed to the more effective resource allocation of the LOF genotypes. As shown in Fig. 6B, although the average available substrate for each cell was similar for both scenarios during the disturbance (solid line in the shaded region), the amount of resources required for producing public goods significantly decreased when an interdependent pattern formed (dashed line), indicating more resources can be assigned to growth or maintenance when the community is facing environment perturbations. This suggests that after the interdependent pattern evolved, the resources that were originally wastefully allocated to produce redundant public goods, were saved to fight against the harsh environmental change. This result can explain why the interdependent pattern is selectively favored at the community level.

## Discussion

In this study, we tested how selfish public goods produced trade-offs that drove the reductive evolution of different interdependent patterns and governed the succession of microbial communities. We found the three classes of interaction patterns that emerged in the steady state communities correlated with different functional traits, including complete functional division, AFC, and one-way dependency. We highlighted the importance of random evolutionary events on the formation of different patterns, where the priority and the relative spatial position of emergence of the different genotypes decide which pair of functional complementary genotypes would dominate the final communities. In addition, communities with these interdependent patterns exhibit a more stable response to disturbance, attributed to the more effective pattern of resource allocation in these communities.

In his work ‘The Origin of species’ [30], Charles Darwin clearly emphasized that natural selection favors the individuals who are selfish to get greatest personal reproductive success, so it was confusing for a long time how cooperation evolved, where an organism is selected to be selfless to enhance the fitness of others [31]. Here, by expanding the BQH framework, our model gives a new insight, that cooperation between auxotrophic genotypes can evolve automatically from the natural selection for selfishness. At the individual-level, LOF is a purely selfish trait, where the individual no longer contributes a public resource to the social group but saves the energy to increase its own reproductive success. However, at the community level, when several functionally complementary LOF genotypes evolve under the right conditions (with proper functional traits), at the right time (emerge simultaneously in the community), and in the right place (spatial proximity to each other), cooperation arises and the newly evolved community possesses the greater selective advantage of better resistance to environmental perturbation, benefiting all members. Thus, in our simulations, the evolution of reciprocal community traits is actually driven by the selection for selfish trait at individual level. This seemingly contradictory idea still follows Darwin’s rule that evolution gives fitness benefit to all the individuals, and gives a possible solution to the paradox of the evolution of cooperation.

Do the interdependent patterns that we observed in our model ever evolve through reductive evolution in nature? A survey based on large-scale metabolic modelling has shown that metabolically interdependent groups are ubiquitous in microbial communities across diverse habitats [32], while further in silico experiments predict that interdependent patterns can arise associated with the production of specific public goods, such as several types of amino acids [33], and the cost of these secretions is a key driver of the mutualistic interactions [34]. Several examples are also reported in experimental systems, one of which is the production of vitamins in marine environments. Although vitamins are indispensable for growth, many plankton species lack a subset of the biosynthetic pathways [23, 35, 36]. This observation is also related to the genome streamlining often seen among oceanic microorganisms [37], because vitamin synthase genes are always energetic costly [38]. A recent study reported that B12 and B1 auxotrophy of the alga *Ostreococcus tauri* could be alleviated by co-culturing with a heterotrophic bacterial partner *Dinoroseobacter shibae*, which in turn relies on the alga to satisfy its requirements of three other B vitamins niacin (B3), biotin (B7), and p-aminobenzoic acid (a precursor for folate, B9) [39]. If we conceptualize these vitamins as five leaky public goods, the two members could be defined as genotype [1, 1, 0, 0, 0] and [0, 0, 1, 1, 1], forming an AFC interdependent pattern as we presented here. It is reasonable to speculate that both genotypes might evolve from their autonomous ancestral populations, respectively, and the selfish vitamin production trade-off drove the reductive evolution. The evolution of interdependent patterns has also been observed in experimental evolution systems. One direct piece of evidence arose from an innovative study by means of a system containing metabolic auxotrophic populations of *Saccharomyces cerevisiae* [40]. The authors found that the strains, originally prototrophic for four metabolites (histidine, leucine, uracil, and methionine) might gradually lose these public functions, when communities with cooperative metabolite exchange began to self-establish. This experimental evolution process exactly matched our simulation results. If we could conduct similar research repeatedly with a longer evolution time, we may experimentally identify the key roles of the function traits, evolutionary randomness, as well as the spatial structure governing the self-establishment of these communities.

Our work also provides a potential explanation for microbial diversity. Leakiness of public goods is widespread within the microbial world, thus co-evolution driven by the sharing of public goods may be common in nature. Considering the complex effects of functional traits and evolutionary randomness, starting from a ‘super ancestor’ who contains multiple leaky functions, diverse LOF species can evolve automatically. Once cooperative interdependent patterns form, genotypic diversity will increase. These strains will share a pan-genome as a public genomic resource, which is identical with the genome of their common “super ancestor” [41]. Since most of these evolved strains are auxotrophic, this idea may also explain why many species are unable to be isolated as pure cultures in the laboratory [42–45].

Different from the previous related model, our model introduced the initial redundancy of public goods production (*β*) as an important parameter to describe functional traits. This assumption allows individuals to overproduce public goods, and should be the prerequisite for the evolution of the LOF genotypes, since they must rely on those redundant public goods for survive. As predicted, we observed that a higher level of the initial degree of functional redundancy was important for the evolution of those interdependent patterns (Fig. 2A). However, accompanied with function loss, the redundancy degree of the whole community decreased. The loss of functional redundancy, or functional distinction, is known as ‘niche complementarity’, where community members occupy complementary niches, buffering environmental perturbation, and conferring gains in productivity and efficiency [46]. This prediction matches with the increased community size (Fig. 2A), as well as increased community resistance (Fig. 6) after the interdependent pattern formed. However, we also found cheaters, who did not contribute to the community, were highly selected for at high levels of redundancy in public goods production, which reduced the community size, i.e., the productivity of the community (Fig. 2B, Top right). Thus, maintaining a proper level of function redundancy to prevent invasion from cheaters may be involved in the design of efficient synthetic communities.

Despite these encouraging insights, our model still has some limitations, which should be addressed in future studies. Firstly, for the sake of simplicity, we assumed that all public functions have equal traits, i.e., equal function costs, degrees of redundancy, and essentiality. However, different public secretions will possess varying function traits. For instance, biosynthesis of different amino acids requires different function costs [18]. The heterogenetic traits of the public functions can potentially lead to divergent community compositions among different interaction patterns. For example, we tested scenarios where the three public functions had different functional redundancy. Although the three types of AFCPs showed similar probability of emergence, the relative abundances among different LOF genotypes, especially the fraction of cheaters ([0, 0, 0]), varied across different patterns (Supplementary Fig. 6). Therefore, we believe that heterogeneity in functional traits should play a role in shaping community structure, and requires further investigation.

Secondly, we did not consider the inherent private benefit of the public goods producer. It has been reported that microbial cells could partially privatize some metabolites, allowing just a fraction of products to leak into the environment, resulting in unequal access to public goods between producer and non-producer [27, 47]. Research has also shown that mutual interdependency can be selectively favored at intermediate levels of privatization, while its absence will lead to the collapse of interaction [27]. Nevertheless, benefiting from our individual-based model, we considered spatial structure (limited mass diffusion), which is also thought to be a vital way for production privatization [29, 48]. Indeed, we observed that a clear concentration gradient of public goods was formed around the related producer cells (Supplementary Fig. 7), suggesting their private benefits. When we removed the spatial structure to perform a simulation in a well-mixed system, we observed that the unlimited expansion of nonproducer genotypes drove community collapse (Supplementary Fig. 8). We also performed simulations including the spatial structure but with varied diffusion coefficients. While the interdependent patterns evolved in a wide range of diffusion rate, increasing the rate of public goods diffusion favored the growth of the non-production cheaters (Supplementary Fig. 9), suggesting higher diffusion level weakened the private benefit of the producers. Our simulations also indicate that lower level of diffusion opposed the evolution of interdependent patterns, because in this scenario, the producers largely privatized the public goods, inhibiting the interactions dependent on public goods sharing. Therefore, private benefits of producers can be derived from both inherent privatization or limited mass diffusion in spatially structured environments, but further studies are still required to discuss the relative contribution of these two factors.

Thirdly, we did not include active cell motility or cell movement via other physical factors (e.g., water flow). In absence of such movement, a daughter cell will be located near its mother cell, resulting in the formation of cell clusters (e.g., microcolony). However, cell movement will break this gathering, where the daughter cell may be soon separated from its mother. Thus, if we added this assumption to our simulations, it may challenge the formation of the segregated interdependent spatial pattern, instead, a pattern with high intermixing of different genotypes may be developed. We believe it would be very interesting to understand the effect of cell movement on the spatial organization of interdependent pattern by combining a new mathematical framework and experimental investigation in future work.

## Methods

### Individual-based model

We used spatial simulations of 2D lattices with periodic boundaries, these were built based on previous studies [11, 12, 49]. A detailed model description is given in Supplementary information S1. Briefly, we used a 100 × 100 array to simulate a spatially structured environment. One microbial individual was allowed to occupy a specific spatial grid box, and could only divide into directly adjacent boxes. We used bit strings to describe the genotypes of microbes as described above (Fig. 1). Public goods and nutrient diffusion were computed using a second-order approximation. Microbial growth is assumed based on the mathematical assumption of the BQH, following the general form:

$$\frac{{dX_i}}{{dt}} \,=\, \left[ {\left( {g_{max,i} - C_i} \right) - \left( {d_{max} - G_i} \right)} \right]X_i,$$

where *X*_*i*_ was the biomass (evaluated by biomass carbon) of the *i*th individual; *g*_*max,i*_ was the maximum growth rate, restricted by a limiting nutrient; *C*_*i*_ was the total cost paid by the individual that performed all functions it carried; *d*_*max*_ was the maximum death rate; *G*_*i*_ was the benefit from the local public goods, as a function of the public goods concentration of the grid box. Public goods diffiusion across the grids was caluculated following a second-order approximation for the 2D diffusion lattice as previously described [49].

Simulations were initialized with ancestral populations [1, 1, 1], and the initial biomass of each individual was set as *X*_0_ = 150 *fg*. Microbes reproduce when biomass reached a upper threshold 2*X*_0_ + *ε*, and died when biomass dropped below a lower threshold 0.2*X*_0_ + *ε*, where *ε* represents uniform random noise in the cell cycle. In addtion, after splitting, the daughter cell is allowed to randomly mutate to lose functions, thus the element ‘1’ in the bit string for the related function may turn into ‘0’ with a certain probability. To charaterize the dynamics of long-term evolution, time-lapse numerical simulations lasted for at least one million time steps, and for a given parameter set, more than 300 repeated simulations were conducted to capture the randomness during the evolution. At each time step, the computation order of the grid boxes is randomized to alleviate the effect of calculation order. All the variables used in the model are listed in Table S1, while all the parameters are provided in Table S2. The model was implemented by C++ language, and the source code are available on (https://github.com/RoyWang1991/Roy-Wang).

### Evolutionary trajectory analysis

During each simulation of evolution dynamics, we specifically made notes of all ‘birth events’ (the first emergence of genotypes), mutation events, and extinction events (the extinction of genotypes). For a given event, the time point, spatial position of occurrence, and the related genotype, as well the genotype of its mother cell, were recorded in detail. These events were regarded as imporant nodes during evolution. Based on these nodes, we established the evolutionary affinities among different genotypes, primarily including what each genotype evolved from and when it evolved or became extinct, forming a tree-like diagram of evolutionary relationships. After trees of all the replicates were built, according to their simlarity, we clustered them manually to draw an overall map of evolutionary trajectories of all the interdependent repeated evolution dynamics (Fig. 3). This analysis was implemented by *Wolfram Mathematica software* (version 10.4).

### Simulations initialized with premixing two AFCPs

To test if the relative PAD at key evolutionary time points had influence on the spatial competition, we initialized communities with four genotypes, [0, 0, 1], [1, 1, 0], [0, 1, 0], and [1, 0, 1], containing two types of AFCPs. We randomly premixed cells of these 4 genotypes at a constant cell number, 3000 cells for the TFLGs ([0, 0, 1] and [0, 1, 0]) and 1500 cells for the OFLGs ([1, 1, 0] and [1, 0, 1]), so that the relative proportion of OFLG and TFLG in an AFCP was roughly in line with the proportion in the final communities of the previous simulations (*α* = 0.001, *β* = 0.8), while the relative abundance of two AFCPs were equal to prevent the effect from differential initial group size. To obtain diverse communities with different relative PADs, 30,000 communities were initialized. The PAI_001:010_ of these communities ranged from 0.93 to 1.07 (Supplementary Fig. 3B), and could be approximately categorized into 15 groups by differential PAI values (i.e., 0.93, 0.94, 0.95…1.07). We then randomly picked a minimum of 40 communities from each group and ran initial simulations with these communities (*α* = 0.001, *β* = 0.8) to steady state (~120,000 time-steps). For a given initial distribution, we conducted 100 replicate simulations to calculate the frequency of winning. The community dynamics, accompanied by the dynamics of PAD values, were analyzed to find the relationship between initial relative PADs and the succession of the communities. These analyses were implemented by *Wolfram Mathematica software* (version 10.4), except for the simulations, which were conducted by modifying the previous C++ code.

### Simulations in well-mixed system

To remove the effect of restricted diffusion of public goods, we conducted simulations in a well-mixed system, where at each time step, public goods and nutrient were rapidly and evenly distributed across the entire array. To realize this setting, at the beginning of each time step, the amount of each substance were summed up and equally spread among the 10^4^ grid boxes, so when calculating microbial growth, there is no concentration difference of public goods and nutrient among the grid boxes. We accomplished this by changing the original setting of substance diffussion in our code.

## Supplementary information

Supplementary video 1

Supplementary video 2

Supplementary video 3

Supplementary video 4

Supplementary video 5

Supporting Information

Supplementary Fig. 1

Supplementary Fig. 2

Supplementary Fig. 3

Supplementary Fig. 4

Supplementary Fig. 5

Supplementary Fig. 6

Supplementary Fig. 7

Supplementary Fig. 8

Supplementary Fig. 9
