# Supplementary material for: Selfishness driving reductive evolution shapes interdependent patterns in spatially structured microbial communities
Source: ISME J. 2020 Dec 20;15(5):1387–401. doi: 10.1038/s41396-020-00858-x (PMC8115099; doi:10.1038/s41396-020-00858-x)
Supplement: Supplementary file 6 — Supporting Information [file 41396_2020_858_MOESM6_ESM.docx]

**Supporting Information**

**Selfishness driving reductive evolution shapes interdependent patterns in spatially structured microbial communities**

Miao-Xiao Wang^1^, Xiao-nan Liu^1^, Yong Nie^1*^, Xiao-Lei Wu^1,2*^

^1^ College of Engineering, Peking University, Beijing 100871, China

^2^ Institute of Ocean Research, Peking University, Beijing 100871, China

*Correspondence: nieyong@pku.edu.cn and xiaolei_wu@pku.edu.cn

**S1 MODEL DESCRIPTION**

Here, we describe our model framework in detail. The model is a spatially-resolved simulation of 2D lattices with periodic boundaries, based on previous work (1-3). The source code for the model framework was written in C++ (https://github.com/RoyWang1991/Roy-Wang). We ran the model on a Ali cloud server running windows. Tables of model variables and parameters are provided in Tables S1 and S2, respectively.

**S1.1 Model initialization**

The model was constructed based on a 100×100 array, where each grid box can only be occupied by one cell. To avoid edge effects in the model, we used periodic boundary conditions which force the array edges to wrap around and meet each other to form a toroidal shape. At the beginning, the concentrations of public goods and nutrient for each grid were set to default values of 0 fg. The autonumous genotype ([1, 1, 1]) cells were randomly assigned to each grid box at default initial frequencies of 0.01 with carbon biomasses of 150 fg (4).

**S1.2 Input, diffusion and loss**

A sole substrate was provided in our model for microbial growth. the dynamics of the substrate in each grid was calculated by

$$\frac{dS_{i}}{dt}=S_{in}+D\cdot\bar{S}_{i}-d_{S}S_{i}-v_{i}X_{i}$$

[1]

Here, $S_{in}$ was the input rate of substrate. We set the substrate input to be spread evenly over every grid at a rate ($S_{in}$) of 1 fg/min/box, to ensure the substrate was not a limiting factor during evolution. Term $D\cdot\bar{S}_{i}$ described the diffusion of substrate across the grids. We applied a second-order approximation for the 2D diffusion lattice as in the previous study (1), where the diffusion rate is proportional to the concentration difference between two adjacent grid boxes. For each lattice element and diffusion step, we calculated the weighted average of the substrate concentration in the 9-element neighbourhood, $\bar{S}_{i}$ (Fig. S1.1). To simulate environments with limited distribution of public goods, we assigned a default diffusion coefficient ($D$) of 0.1 fg/fg∙min, according to published diffusion coefficients for several organic compounds (5). With a time step of 1 min, this coefficient meant concentrations of substrate in two grid boxes could not reach equilibrium in one time step, resulting in a diffusion limitation. We also performed simulations to test the effect of $D$ (its value ranged from 0.01 to 0.5) on the evolution of interdependent patterns (Supplementary Figure 9). In addition, $d_{S}$ was the abiotic degradation rate of the substrate, whose default value was 0.01 min^-1^ (5), and $v_{i}$ was the uptake rate of the substrate by an individual, whose calculation is described in the next part (equation [4], **S1.3.1**).

**S1.3 Microbial growth associated with public goods dynamics**

We assumed a microbial growth rate based on the basic mathematical assumption of the BQH, which combined the benefit and cost of function loss to calculate the microbial growth rate. Using this assumption, for a specific LOF individual in the *ith* grid, the biomass growth was given by

$$\frac{dX_{i}}{dt}={(g}_{i}-d_{i})X_{i}$$

[2]

where $X_{i}$ was the biomass of the individual;$g_{i}$ was the growth rate, definded as the maximum growth rate, $g_{max,i}$, minus the total cost paid by the individual that performed all functions it carried, $C_{i}$, so that,

$$g_{i}=g_{max,i}-C_{i}$$

[2]

$d_{i}$ was the death rate, given by the difference between maximum death rate,$d_{max}$, and the benefit from the local public goods, $G_{i}$, so that

$$d_{i}=d_{max}-G_{i}$$

[3]

According to the equation [2], a genotype that performed more functions would grow slower, so $C_{i}$ characterized the fitness benefit of the individual after function loss. In addition, $G_{i}$ was positively related with the concentration of local public goods, thus the individual would decline faster when the grid was absent of the public goods. Therefore, $G_{i}$ acted as a measure of fitness cost for function loss.

**S1.3.1 Calculation of intrinsic specific growth rate** $\boldsymbol{g}_{\boldsymbol{max,i}}$

The intrinsic specific growth rate $g_{max,i}$ was restricted by the uptake of the sole nutrient. For the individual in the *ith* grid, substrate uptake rate $v_{i}$ followed the classical Michaelis-Menten kinetics,

$$v_{i}=\frac{V_{max}*S_{i}}{K_{m}+S_{i}}$$

[4]

where $V_{max}$= 0.0461 fg/fg∙min was the maximum rate of uptake at product saturation, and $K_{m}$= 2.34×10^−3^ fg is the half-saturation constant. These values fall within the ranges reported by (6). After nutrient uptake, microbial metabolism converts the uptake nutrient into biomass at a fixed efficiency given by the yield constant, $Y_{max}$= 0.4444 fg/fg (1), thus $g_{max,i}$ could be calculated by,

$$g_{max,i}=v_{i}Y_{max}=\frac{Y_{max}\cdot V_{max}\cdot S_{i}}{\left( K_{m}+S_{i} \right)}$$

[5]

**S1.3.2 Public goods production, consumption and loss**

In our model, we simply assumed at each time step, an individual who performed the function *j* would used a fraction, $\alpha_{j}$, of its biomass to produce public good *j*, thus the production rate of public good *j* was

$$p_{i,j}=\alpha_{j}X_{i}$$

[6]

When public goods were produced, they were directly secreted to the grid, neglecting any step of transport from intracelluar to the enviroment. We assumed this production was redundant for microbial demand. In other words, producers overproduced the public goods, where the redundant products provided an assurance that individuals could survive when public goods are diluted by diffusion or loss. To conceptualize this redundancy, we assumed that the required amount of public goods in each step accounted for the produced public goods with a ratio of $\beta_{j}$. Therefore, the redundant fraction of public goods production was $1-\beta_{j}$ and lower $\beta_{j}$ would reflect larger redundancy degree of function *j*. Accordingly, the public goods consumed at each time step,${CP}_{i,j}$, relied on the concentration of the public good *j* in the grid, $P_{i,j}$,

$${CP}_{i,j}=\left\{ \begin{aligned} \beta_{j}\alpha_{j}X_{i} when P_{i,j}>\beta_{j}\alpha_{j}X_{i} \\ P_{i,j} when P_{i,j}<\beta_{j}\alpha_{j}X_{i} \end{aligned} \right.$$

[7]

Accompanied by abiotic loss at a rate of $d_{P}$ (default value is 0.01 min^-1^), the dynamics of public goods concentration in a grid,$P_{i,j}$ would be given by

$$\frac{dP_{i,j}}{dt}=p_{i,j}-{CP}_{i,j}-d_{P}P_{i,j}$$

[8]

In addition, after production and utilization in each step, public goods diffuse across the grids calculated according to part S1.2, with default diffusion coefficient ($D$) of 0.1 fg/fg∙min.

**S1.3.3 The fitness benefit associated with public goods production**

According to the BQH, the fitness benefit of the individual who lost a public function, is identical to the cost ($C_{i}$) of the limiting resource for a producer to perfom the function (7). This cost can be divided into two parts: 1) resources directly used for producing public goods; 2) extra resource cost associated with the synthesis of public goods (i.e., substrate cannot be completely converted to public goods). The first part is equal to $p_{i,j}$. For the second part, a constant rate for extra cost, $e_{j}$ = 10 % was given to consider the extra resource loss. Therefore, for a given individual of genotype [$x_{1}, x_{2}, x_{3}$] (where $x_{j}$= 1 or 0, denoted preforming or not the related function), its total cost of performing public function, $C_{i}$, could be computed by

$$C_{i}=\sum_{j}^{3} x_{j}\left( 1+e_{j} \right)\alpha_{j}X_{i,t}$$

[9]

Accoding this equation, $\alpha_{j}$ could directly reflect the function cost of the function *j*.

**S1.3.4 The fitness cost (**$\boldsymbol{G}_{\boldsymbol{i}}$**) associated with public goods concentration around**

The fitness cost can be generated from the decreased activity when the public good under consideration was not sufficient (7). The public goods considered here were all strictly essential, so we proposed three assumptions in our model: 1) when the amout of a public goods in a grid was less than the required, $\beta_{j}\alpha_{j}X_{i}$, the benefit of the related individual from the local public goods, $G_{i}$, would reduce, so its death rate would be increase according to equation [3]; 2) in absence of any public good, the biomass of a individual would decline, which means that $G_{i}$= 0 and $d_{max}>g_{max,i}$; 3) when all public goods were sufficient, the death rate of a individual would equal to its intrinsic death rate, defined as $d_{min}$. Accordingly, the fitness cost, $G_{i}$, is computed by,

$$G_{i}=\frac{\prod_{j=1}^{3} \frac{\left( d_{max}-d_{min} \right){CP}_{i,j}}{\beta_{j}\alpha_{j}X_{i}}}{{(d_{max}-d_{min})}^{2}}$$

[10]

In summary, the growth dynamics of the individual in the *ith* grid follows:

$$\frac{dX_{i}}{dt}=\left\{ \left[ \frac{Y_{max}\cdot V_{max}\cdot S_{i}}{\left( K_{m}+S_{i} \right)}-\sum_{j}^{3} x_{j}\left( 1+e_{j} \right)\alpha_{j}X_{i,t} \right]-\left[ d_{max}-\frac{\prod_{j=1}^{3} \frac{\left( d_{max}-d_{min} \right){CP}_{i,j}}{\beta_{j}\alpha_{j}X_{i}}}{{(d_{max}-d_{min})}^{2}} \right] \right\})X_{i}$$

[11]

Let

$$d_{max}=Y_{max}\cdot V_{max}\geq g_{max,i}$$

so when any single public good was absent, that is, $P_{i,j}=0$ and ${CP}_{i,j}$ = 0, then $\frac{dX_{i}}{dt}<0$, thus ensure its essentiality. Alternatively, if all public goods were sufficient, that is, $P_{i,j}>\beta_{j}\alpha_{j}X_{i}$ and ${CP}_{i,j}$=$\beta_{j}\alpha_{j}X_{i}$, then $d_{i}$ equals the intrinsic death rate, $d_{min}$, whose default value is 10^-3^ min^-1^.

**S1.4 Microbial processes**

When the carbon biomass of a individual reached a upper threshold, $X_{i,max}$ = $(2+\varepsilon)X_{0}$, it divided into two cells with equal mass.$X_{0}$ represents initial biomass, whose default value is 150 fg, and $\varepsilon$ represents uniform random noise in the cell cycle, follows a uniform distribution. The mother cell remained in the original grid box, while the daughter cell randomly moved to one of the 9 directly adjacent grids. If the randomly chosen box is already occupied by another cell, the daughter cell competes with the original occupant on an equal level, where one of the cells dies at random with probability 0.5.

During the division, mutations occurr at a rate of *mut* = 10^−5^/function/division in the daughter cell. If a function loss occurs, the genotype of the daughter cell is changed, where the element ‘1’ in the bit string for the related function turns into ‘0’. For all three functions, mutation occur independently. Our model did not include function restoration or horizontal gene transfer, because our goal was to consider how the Black Queen evolution affects the community dynamics, but it would be easy to add these assumptions to our model framework.

Microbial death randomly occurred at a rate of *de*=1×10^−4^/min, as well as when C biomass of an individual decayed below $X_{i,max}$ = $(0. 2+\varepsilon)X_{0}$, which was based on the low end of bacterial cell sizes (4). When a microbe died, its biomass was added back to the grid directly as nutrient without any degradation processes.

**S1.5 Iteration**

While abiotic and biotic processes occur simultaneously in natural ecosystems, , in computional simulations they must be computed sequentially across all grid boxes. The computation order of the grids was randomized to decrease the effect of calculation order.

**Figures**

**
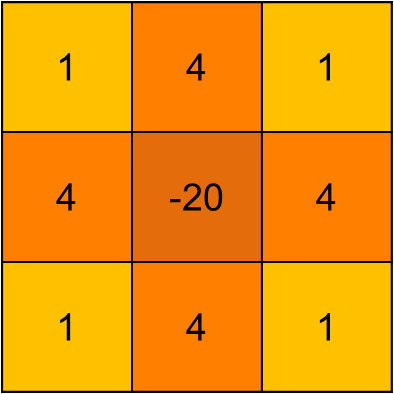
**

**Fig. S1.1** Weights in the 9-element neighbourhood for diffusion computation.

**S2 TABLE OF SIMBOLS**

**Table S1 Summary of model variables**

| **Symbol** | **Definition** | **Units** |
| --- | --- | --- |
| ${[x_{1}, x_{2}, x_{3}]}_{i}$ | Genotype of the individual in the *ith* grid. |  |
| $X_{i}$ | Carbon biomass of the individual in the *ith* grid. | fg |
| $X_{i}(U)$ | Upper threshold of C biomass of an individual. | fg |
| $X_{i}(L)$ | Lower threshold of C biomass of an individual. | fg |
| $g_{i}$ | The growth rate of the individual in the *ith* grid. | fg/fg∙min |
| $d_{i}$ | The death rate of the individual in the *ith* grid. | fg/fg∙min |
| $g_{max,i}$ | The maximum growth rate | fg/fg∙min |
| $C_{i}$ | Total cost paid by the individual that performed all functions it carried. | fg/fg∙min |
| $G_{i}$ | the benefit from the local public goods |  |
| $S_{i}$ | Substrate concentration in the *ith* grid. | fg |
| $\bar{S}_{i}$ | Weighted average of all substrate concentration in the 9-element neighbourhood around the *ith* grid. | fg |
| $v_{i}$ | Nutrient uptake rate the individual in the *ith* grid at the time step *t*. | fg/fg∙min |
| $p_{i,j}$ | Production rate of public good *j* | fg/min |
| $P_{i,j}$ | Amount of public good *j* in the *ith* grid. | fg |
| ${CP}_{i,j}$ | The amount of public good *j* consumed by the *ith* individual at each time step. | fg∙min |

**Table S2 Summary of model parameters**

| **Symbol** | **Definition** | **Units** | **Values** | **References** |
| --- | --- | --- | --- | --- |
| $d_{max}$ | The maximum death rate | fg/fg∙min | 0.02 | (1) |
| $d_{min}$ | The minimal death rate | fg/fg∙min | 0.001 | (2) |
| $S_{i,0}$ | Initial substrate concentration of each grid. | fg | 0 |  |
| $S_{in}$ | Substrate concentration input to each grid. | fg ∙min | 1 | (2) |
| $d_{S}$ | The abiotic degradation rate of the substrate. | fg/fg∙min | 0.01 | (2) |
| $d_{P}$ | The abiotic degradation rate of the public good *j.* | fg/fg∙min | 0.01 | (2) |
| $e_{j}$ | Rate for extra resource cost of public good *j*. |  | 10 % | (1) |
| $X_{i,0}$ | Initial C biomass of an individual. | fg | 150 | (4) |
| $\varepsilon$ | Uniform random noise in the cell cycle |  | U (-0.2, 0.2) | (8) |
| $D$ | Diffusion coefficient. | fg/fg∙min | 0.1 | (5) |
| $V_{max}$ | Maximum rate of uptake at product saturation | fg/fg∙min | 0.0461 | (6) |
| $K_{m}$ | Half-saturation constant | fg/µm^2^ | 2.34×10^−3^ | (6) |
| $Y_{max}$ | Efficiency of substrate conversion to biomass | fg/fg | 0.4444 | (5) |
| $\alpha_{j}$ | The fraction of biomass used to produce public good *j* per unit time of an individual*.* | fg/fg·min | 1e-4, 5e-4, 1e-3 | (7) |
| $\beta_{j}$ | The ratio of the required amount of public good *j* accounting for the produced public good *j.* |  | 0.4, 0.6, 0.8 | (1) |
| *mut* | Mutation rate. | /function·division | 10^−5^ | (1) |
| *de* | random death rate | /min | 1×10^−4^ | (2) |

**S3 SUPPLEMENTARY FIGURES AND VIDEOS Legends**

**Supplementary Fig. 1** The time gap between the emergence of the second one function loss genotype and the extinction of the autonomous genotype [1, 1, 1] across different combinations of function traits. A longer time gap indicates the slower extinction processes for [1, 1, 1] after the second one function loss genotype emerged, resulting in a higher chance for cheaters [0, 0, 0] to meet [1, 1, 1], forming the one-way dependency pattern.

**Supplementary Fig. 2** α-diversity dynamics during the evolution processes across gradients of function cost (α) and functional redundancy (1-β). Different clusters of paths are shown by different colors (figure label at right). Dashed box indicates the replicates where one type of asymmetric functional complementary pair dominated the final communities, but succeed via different modes. Trend analyses are shown by curve, using *R* programming, package *ggplot2*.

**Supplementary Fig. 3** (A) The weight of adjacent partners when calculating ‘partner association degree’ and examples of calculations; (B) Distribution of partner association degree of 30000 communities randomly premixed with [0, 0, 1] + [1, 1, 0] + [0, 1, 0] + [1, 0, 1]. The ratio of premixing was 1500 : 3000 : 1500 : 3000 for the four genotypes. The distribution matches a Gaussian distribution (*N* (1.00, 0.02), R^2^ = 0.9996, P < 0.05).

**Supplementary Fig. 4** Dynamics of the association degree of the winning asymmetric functional complementary pairs (AFCPs) when conducted simulations were initialized with two AFCPs [0, 0, 1] & [1, 1, 0] and [0, 1, 0] & [1, 0, 1]. PAD_win_ was the PAD values of the winning AFCPs. Three groups of simulations with different initial PAI_001:010_ values were shown to represent three typical scenarios where pair [0, 0, 1] & [1, 1, 0] were more, similar, or less associated, than pair [0, 1, 0] & [1, 0, 1], respectively. PAD_win_ was increased from approximately 3.0 (random mix) to 4.7 (interdependent spatial aggregate formed) and keep stable.

**Supplementary Fig. 5** Other random events may contribute to, but, not change the odds of winning for an asymmetric functional complementary pair (AFCP). (A) Simulations are starting from a symmetric spatial pattern in which two AFCPs [0, 0, 1] & [1, 1, 0] and [0, 1, 0] & [1, 0, 1] had an identical association degree and group size. (B) The segregated interdependent spatial pattern still emerged, and one AFCP would outcompete the other, dominating the final community in each run. (C) In 100 repeated runs, the final communities were equiprobably dominated by the two AFCPs.

**Supplementary Fig. 6** The final (steady state) community structures when the three public functions had different functional redundancy. Results were summarized from 234 independent runs. Community structures were assessed at 1700000 min, when all runs had reached steady state. Running conditions: *mutation rate* = 10^-5^, α = 0.001, and three distinct functional redundancies: β_1_ = 0.8, β_2_ = 0.6, β_3_ = 0.4.

**Supplementary Fig. 7** The community dynamics when conducting simulations in a well-mixed system. Runs were representative examples from 300 runs for the two kinds of treatments. Running conditions: α = 0.001, β = 0.8; *mut* = 10^-5^ for the simulations initial with [1, 1, 1] (first line); *mut* = 0 for the simulations initialized with two AFCPs (second line).

**Supplementary Fig. 8** Community dynamics associated with public goods distribution dynamics on the spatial lattices. A representative example in keeping with Fig. 2 line 4 is shown. Note the concentration gradient formed around the related public goods producers. For example, the concentration gradient of public goods 1 and 2 formed around [1, 1, 0] in the final community (1700000 min). The initial public goods concentrations were zero, thus not shown.

**Supplementary Fig. 9** The effects of diffusion rate on the evolution of interdependent patterns. The final (steady state) community structures across a gradient of diffusion coefficient, $D$. Results of each condition was summarized from 300 independent runs. Community structures were assessed at 1700000 min, when all runs had reached steady state. Running conditions: *mutation rate* = 10^-5^, α = 0.001, β = 0.8.

**Supplementary video 1** The evolution of asymmetric functional complementary pattern between one function loss genotype [0, 1, 1] and two function loss genotype [1, 0, 0] through a simple evolutionary path. After the emergence of [1, 0, 0], it formed a spatial aggregate with its functional complementary partner [0, 1, 1]. This aggregate rapidly expanded and dominated the community, and no other asymmetric functional complementary pair emerged during the expansion process. This is the same replicate as the first row of Figure 2A.

**Supplementary video 2** The evolution of asymmetric functional complementary pattern between one function loss genotype [1, 0, 1] and two function loss genotype [0, 1, 0] through a simple evolutionary path. After the emergence of [0, 1, 0], it formed a spatial aggregate with its functional complementary partner [1, 0, 1]. This aggregate rapidly expanded and dominated the community, and no other asymmetric functional complementary pair emerged during the expansion process. This is the same replicate as the second row of Figure 2A.

**Supplementary video 3** The evolution of asymmetric function complementary pattern between one function loss genotype [1, 1, 0] and two function loss genotype [0, 0, 1] through a simple evolutionary path. After the emergence of [0, 0, 1], it formed a spatial aggregate with its functional complementary partner [1, 1, 0]. This aggregate rapidly expanded and dominated the community, and no other asymmetric functional complementary pair emerged during the expansion process. This is the same replicate as the third row of Figure 2A.

**Supplementary video 4** The evolution of asymmetric function complementary pattern between one function loss genotype [1, 1, 0] and two function loss genotype [0, 0, 1] through spatial competition with another asymmetric functional complementary pair (AFCP). After the emergence of [0, 0, 1], it formed a spatial aggregate with its functional complementary partner [1, 1, 0]. However, another AFCP ([0, 1, 0] and [1, 0, 1]) also formed an aggregate. These aggregates self-organized into a segregated spatial pattern, resulting in a spatial competition between them. Finally, AFCP [0, 0, 1] and [1, 1, 0] won the competition and dominated the final community. This is the same replicate as the fourth row of Figure 2A.

**Supplementary video 5** The evolution of asymmetric function complementary pattern between one function loss genotype [1, 1, 0] and two function loss genotype [0, 0, 1] through a spatial competition with two other asymmetric functional complementary pair (AFCPs). After the emergence of [0, 0, 1], it formed a spatial aggregate with its functional complementary partner [1, 1, 0]. However, two other AFCPs, [0, 1, 0] + [1, 0, 1] and [1, 0, 0] + [0, 1, 1], also formed aggregates. These aggregates self-organized to a segregated spatial pattern, where a spatial competition took place between them. In the end, AFCP [0, 0, 1] and [1, 1, 0] won the competition and dominated the final community. This is the same replicate as the fifth row of Figure 2A.

**SI REFERENCES**

1. Kreft JU, Booth G, & Wimpenny JW (1998) BacSim, a simulator for individual-based modelling of bacterial colony growth. *Microbiology* 144 ( Pt 12):3275-3287.

2. Allison SD (2005) Cheaters, diffusion and nutrients constrain decomposition by microbial enzymes in spatially structured environments. *Ecology Letters* 8(6):626-635.

3. Folse HJ & Allison SD (2012) Cooperation, competition, and coalitions in enzyme-producing microbes: social evolution and nutrient depolymerization rates. *Front Microbiol* 3.

4. Button DK (1998) Nutrient uptake by microorganisms according to kinetic parameters from theory as related to cytoarchitecture. *Microbiol Mol Biol Rev* 62(3):636-645.

5. Vetter YA, Deming JW, Jumars PA, & Krieger-Brockett BB (1998) A Predictive Model of Bacterial Foraging by Means of Freely Released Extracellular Enzymes. *Microb Ecol* 36(1):75-92.

6. Koch AL & Wang CH (1982) How Close to the Theoretical Diffusion Limit Do Bacterial Uptake Systems Function. *Arch Microbiol* 131(1):36-42.

7. Morris JJ, Lenski RE, & Zinser ER (2012) The Black Queen Hypothesis: evolution of dependencies through adaptive gene loss. *MBio* 3(2).

8. Rudge TJ, Steiner PJ, Phillips A, & Haseloff J (2012) Computational modeling of synthetic microbial biofilms. *ACS Synth Biol* 1(8):345-352.
