# Supplementary figures and images for: Selfishness driving reductive evolution shapes interdependent patterns in spatially structured microbial communities
Source: ISME J. 2020 Dec 20;15(5):1387–401. doi: 10.1038/s41396-020-00858-x (PMC8115099; doi:10.1038/s41396-020-00858-x)

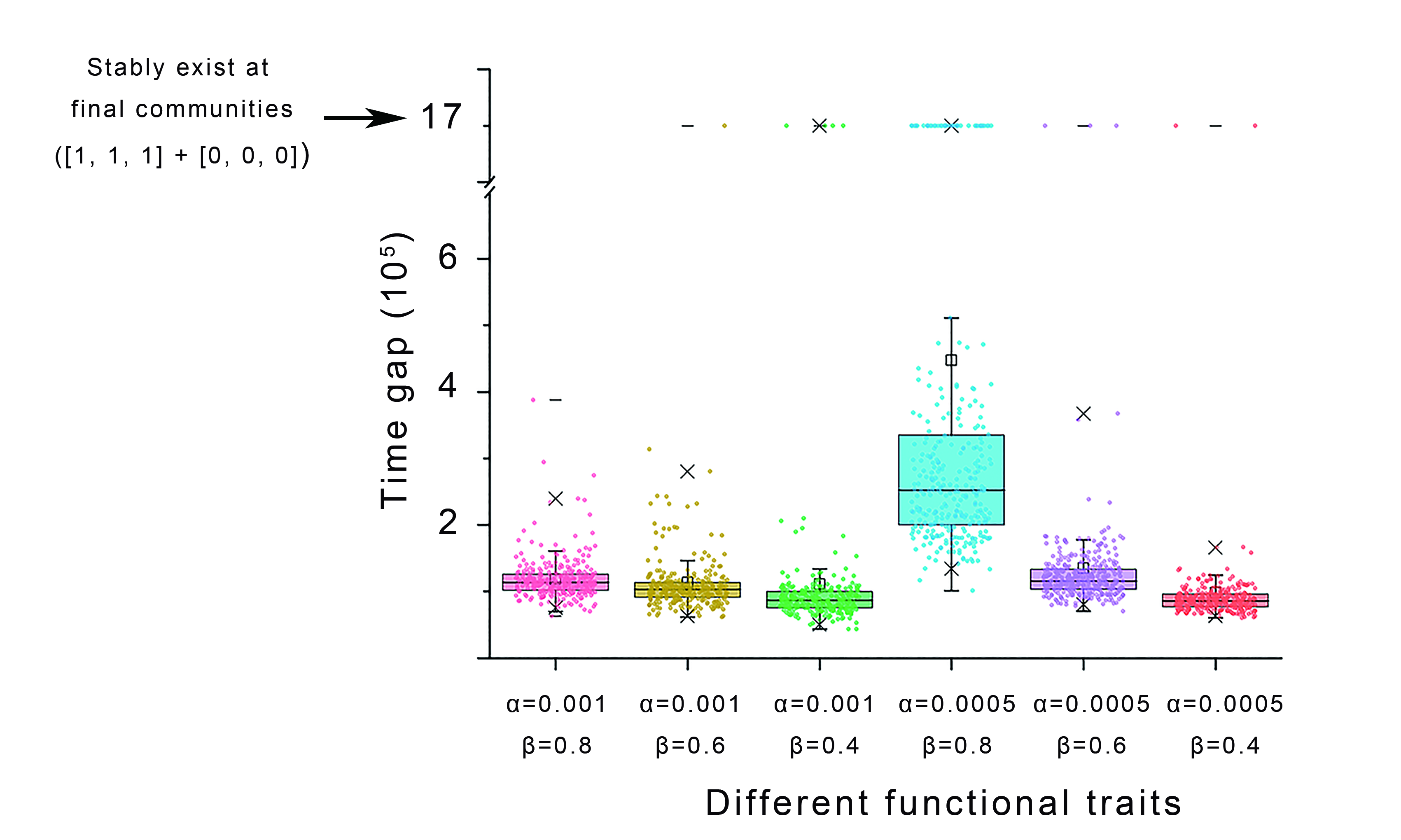

Supplement: Supplementary file 7 — Supplementary Fig. 1 [file 41396_2020_858_MOESM7_ESM.tif]

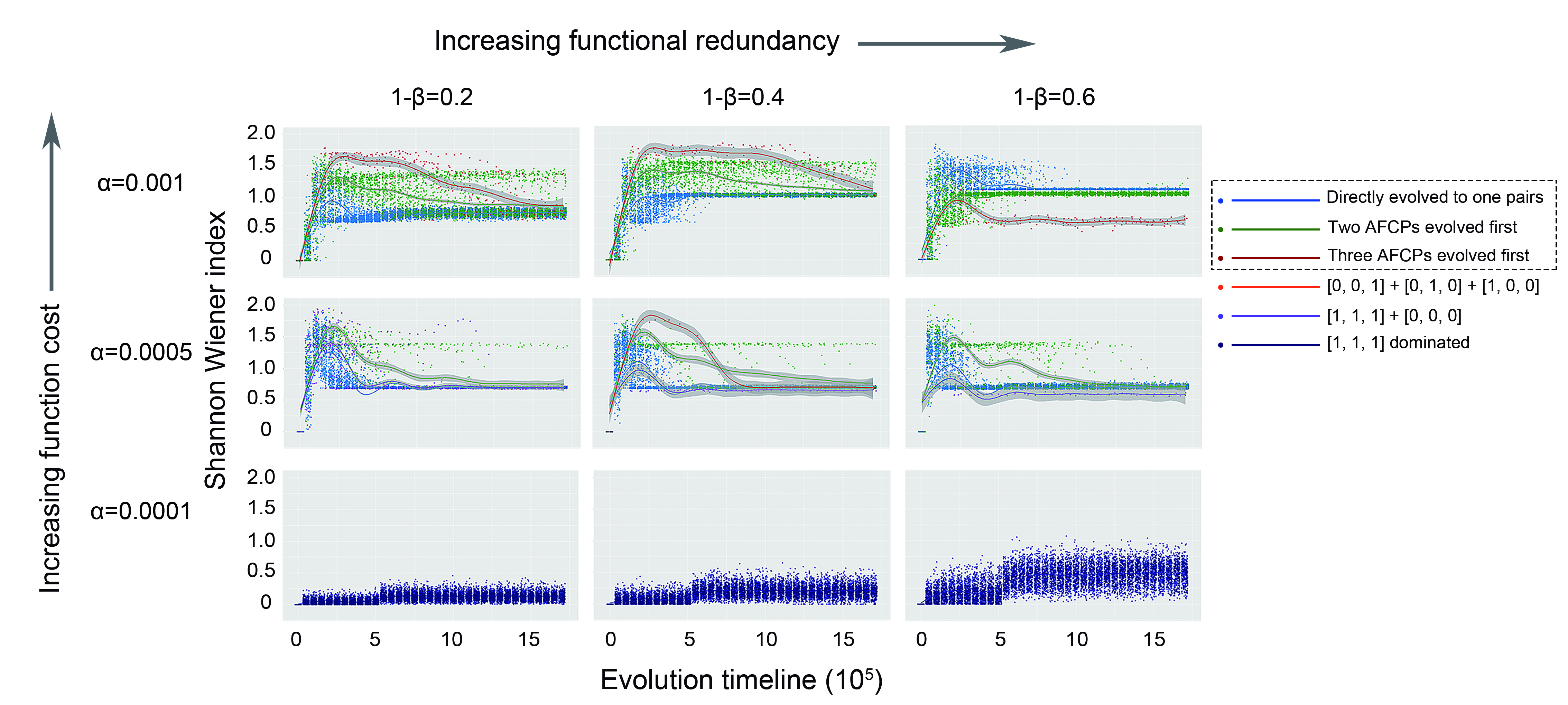

Supplement: Supplementary file 8 — Supplementary Fig. 2 [file 41396_2020_858_MOESM8_ESM.tif]

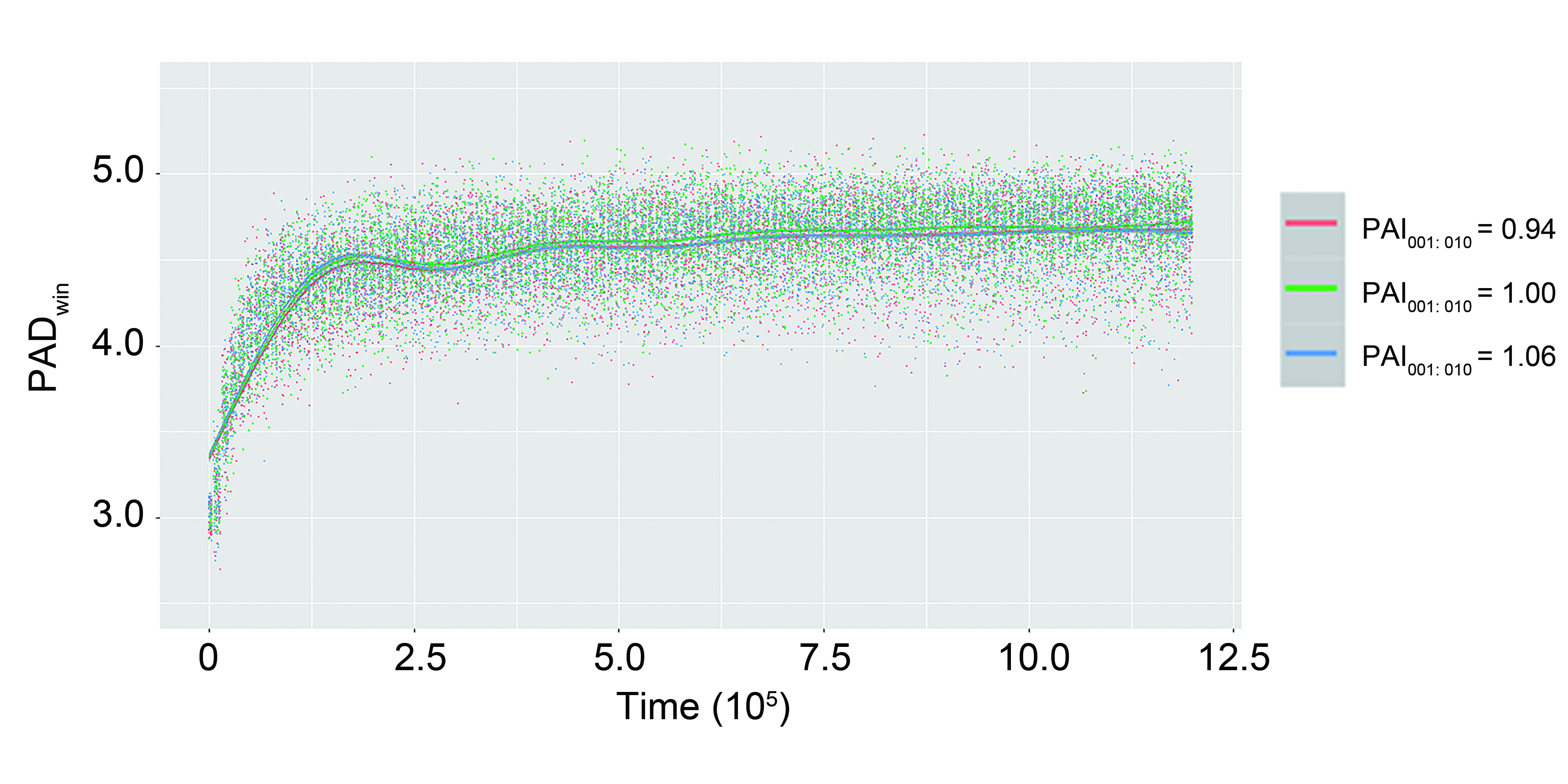

Supplement: Supplementary file 10 — Supplementary Fig. 4 [file 41396_2020_858_MOESM10_ESM.tif]

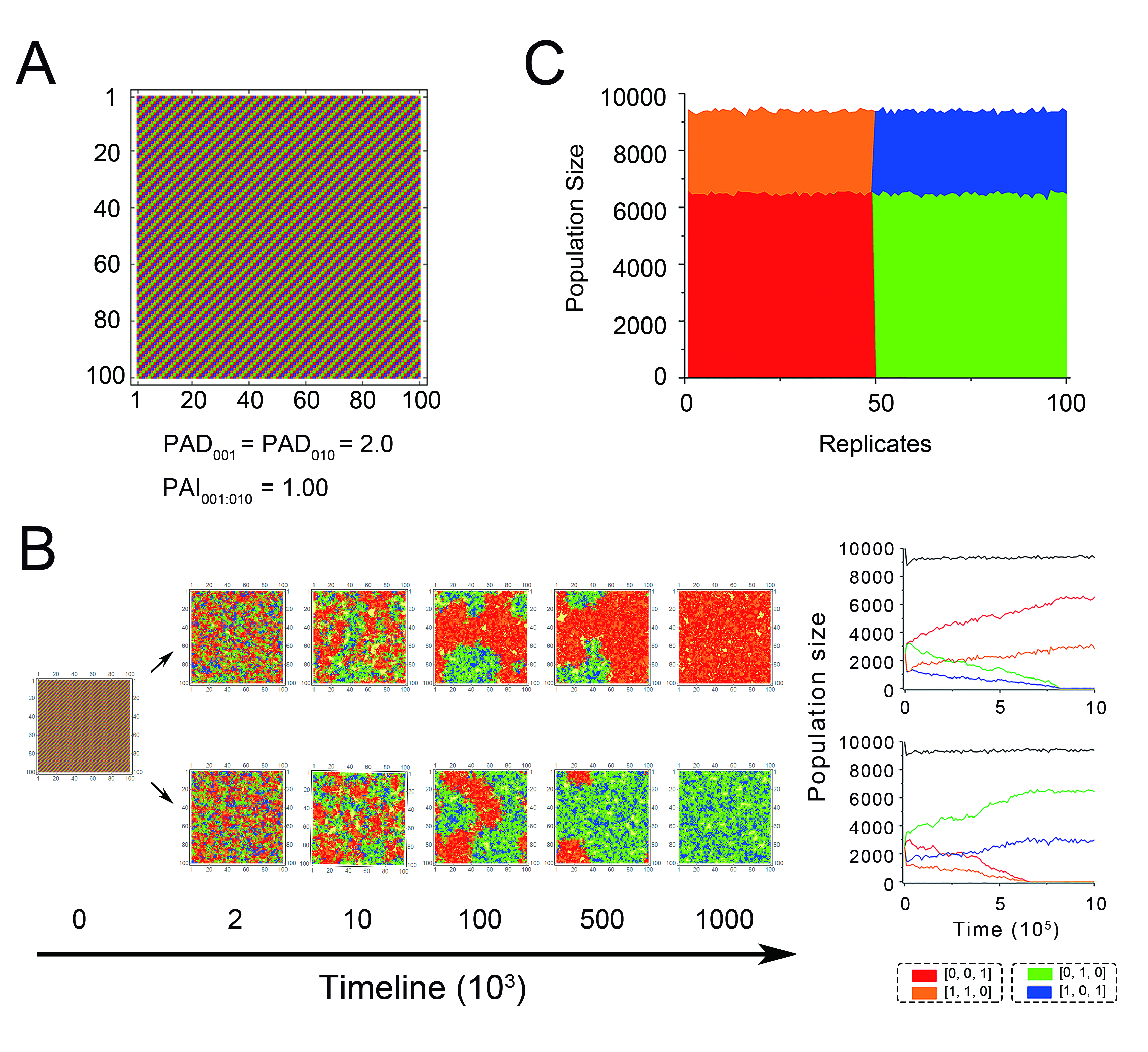

Supplement: Supplementary file 11 — Supplementary Fig. 5 [file 41396_2020_858_MOESM11_ESM.tif]

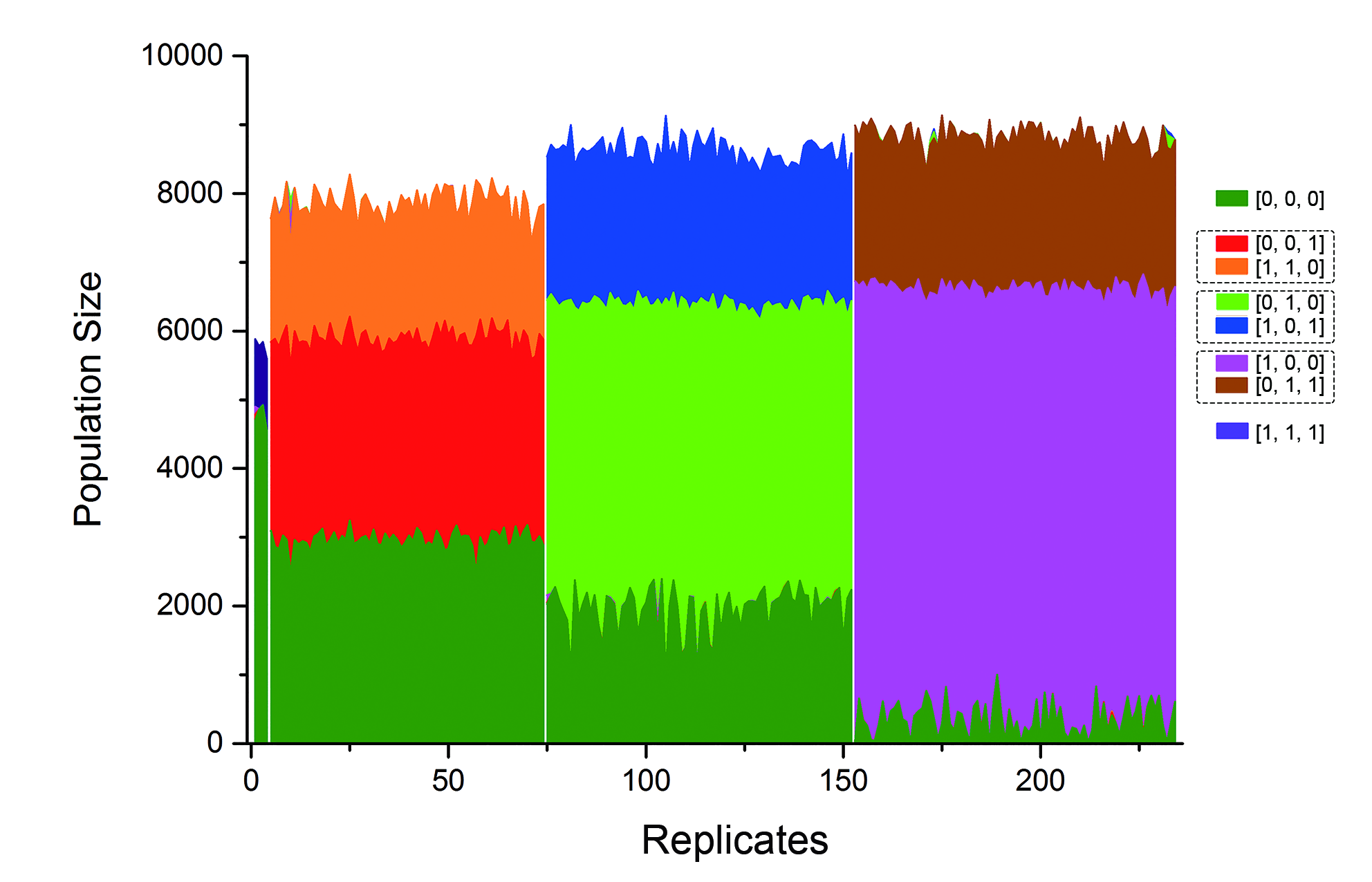

Supplement: Supplementary file 12 — Supplementary Fig. 6 [file 41396_2020_858_MOESM12_ESM.tif]

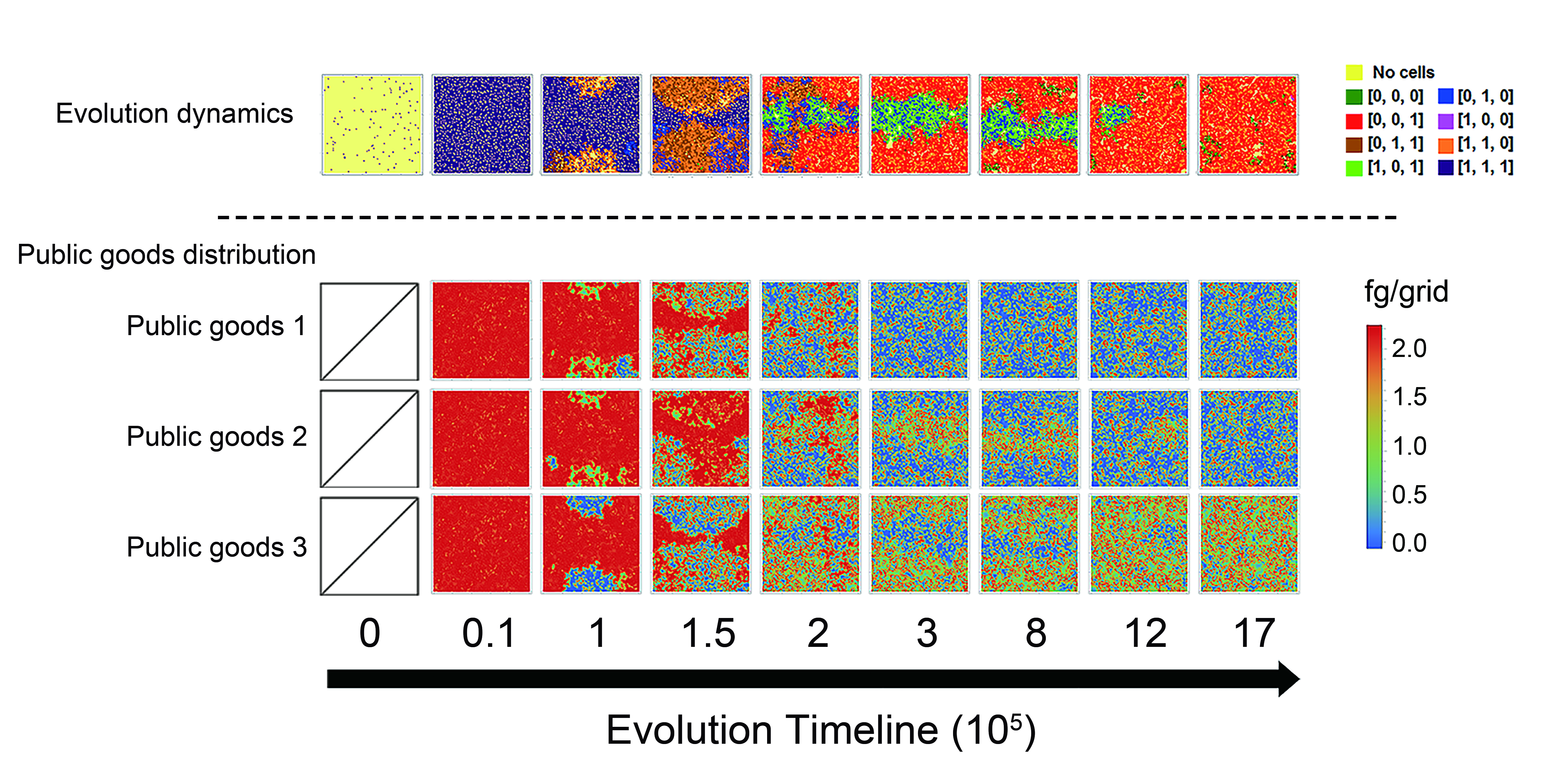

Supplement: Supplementary file 13 — Supplementary Fig. 7 [file 41396_2020_858_MOESM13_ESM.tif]

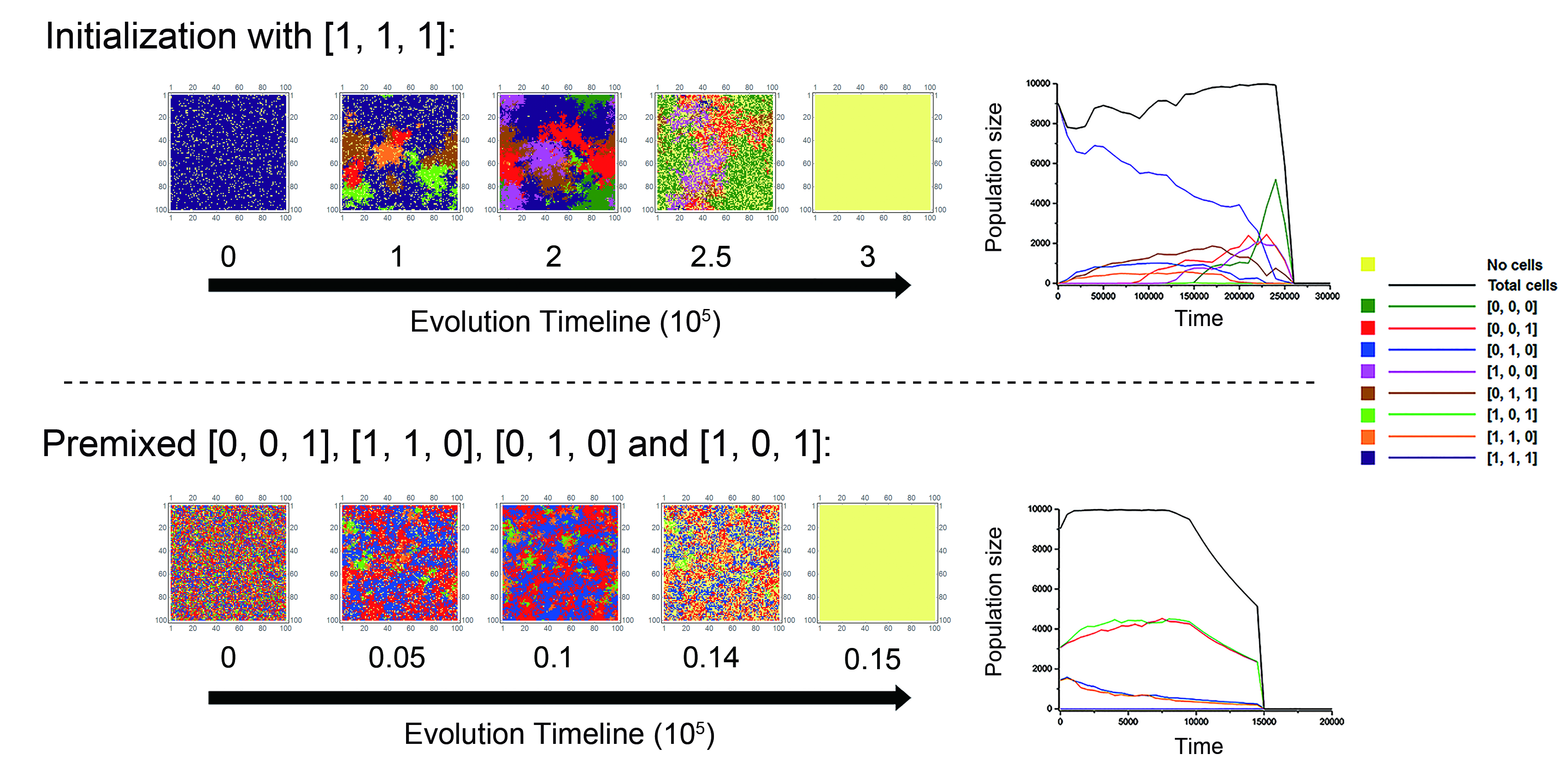

Supplement: Supplementary file 14 — Supplementary Fig. 8 [file 41396_2020_858_MOESM14_ESM.tif]

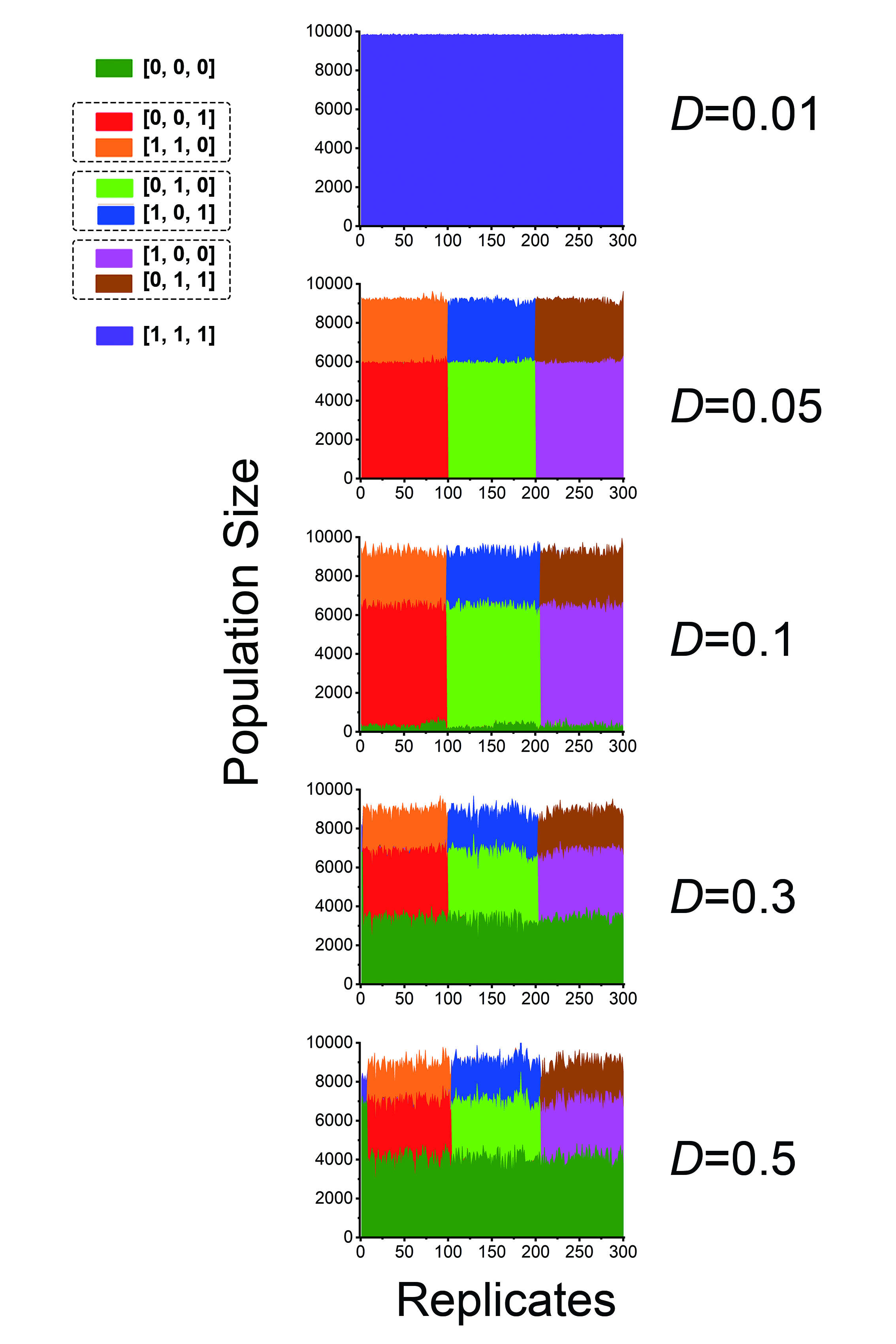

Supplement: Supplementary file 15 — Supplementary Fig. 9 [file 41396_2020_858_MOESM15_ESM.tif]
